# Translation suppression underlies the restrained COVID-19 mRNA vaccine response in the high-risk immunocompromised group

**DOI:** 10.3389/fimmu.2022.1020165

**Published:** 2022-10-26

**Authors:** Kangsan Kim, Madhusudhanan Narasimhan, Lenin Mahimainathan, Ray Zhang, Ellen Araj, Elizabeth Kim, William Tharpe, Benjamin M. Greenberg, David E. Greenberg, Quan-Zhen Li, Chi-An Cheng, Ravi Sarode, Srinivas Malladi, Alagarraju Muthukumar

**Affiliations:** ^1^ Department of Pathology, The University of Texas Southwestern Medical Center, Dallas, TX, United States; ^2^ William P. Clements Jr. University Hospital, The University of Texas Southwestern Medical Center, Dallas, TX, United States; ^3^ Department of Neurology and Neurotherapeutics, The University of Texas Southwestern Medical Center, Dallas, TX, United States; ^4^ Department of Internal Medicine, The University of Texas Southwestern Medical Center, Dallas, TX, United States; ^5^ Department of Microbiology, The University of Texas Southwestern Medical Center, Dallas, TX, United States; ^6^ Microarray Core, Department of Immunology, The University of Texas Southwestern Medical Center, Dallas, TX, United States; ^7^ School of Pharmacy, National Taiwan University, Taipei, Taiwan

**Keywords:** covid-19 mRNA vaccines, translation suppression, immunocompromised patients, sirolimus, mycophenolate, tacrolimus

## Abstract

**Background:**

Immunocompromised (IC) patients show diminished immune response to COVID-19 mRNA vaccines (Co-mV). To date, there is no ‘empirical’ evidence to link the perturbation of translation, a rate-limiting step for mRNA vaccine efficiency (VE), to the dampened response of Co-mV.

**Materials and methods:**

Impact of immunosuppressants (ISs), tacrolimus (T), mycophenolate (M), rapamycin/sirolimus (S), and their combinations on Pfizer Co-mV translation were determined by the Spike (Sp) protein expression following Co-mV transfection in HEK293 cells. *In vivo* impact of ISs on SARS-CoV-2 spike specific antigen (SpAg) and associated antibody levels (IgG_Sp_) in serum were assessed in Balb/c mice after two doses (2D) of the Pfizer vaccine. Spike Ag and IgG_Sp_ levels were assessed in 259 IC patients and 50 healthy controls (HC) who received 2D of Pfizer or Moderna Co-mV as well as in 67 immunosuppressed solid organ transplant (SOT) patients and 843 non-transplanted (NT) subjects following three doses (3D) of Co-mV. Higher Co-mV concentrations and transient drug holidays were evaluated.

**Results:**

We observed significantly lower IgG_SP_ response in IC patients (p<0.0001) compared to their matched controls in 2D and 3D Co-mV groups. IC patients on M or S showed a profound dampening of IgG_SP_ response relative to those that were not on these drugs. M and S, when used individually or in combination, significantly attenuated the Co-mV-induced Sp expression, whereas T did not exert significant influence. Sirolimus combo pretreatment *in vivo* significantly attenuated the Co-mV induced IgM_Sp_ and IgG_Sp_ production, which correlated with a decreasing trend in the early levels (after day 1) of Co-mV induced Sp immunogen levels. Neither higher Co-mV concentrations (6μg) nor withholding S for 1-day could overcome the inhibition of Sp protein levels. Interestingly, 3-days S holiday or using T alone rescued Sp levels *in vitro.*

**Conclusions:**

This is the first study to demonstrate that ISs, sirolimus and mycophenolate inhibited Co-mV-induced Sp protein synthesis *via* translation repression. Selective use of tacrolimus or drug holiday of sirolimus can be a potential means to rescue translation-dependent Sp protein production. These findings lay a strong foundation for guiding future studies aimed at improving Co-mV responses in high-risk IC patients.

## Introduction

Vaccines of different classes have been approved and in use to combat Coronavirus disease 2019 (COVID-19). Among them, the non-replicating mRNA vaccines, BNT162b2 (Pfizer–BioNTech) and mRNA-1273 (Moderna) that encode Severe Acute Respiratory Syndrome Coronavirus 2 (SARS-CoV-2) spike (Sp) protein’s receptor binding domain (RBD) have proven to be highly effective across patient populations worldwide. Several promising mRNA candidates are also in development for COVID-19 and other clinical conditions. However, we and others have reported that COVID-19 mRNA vaccines (Co-mV) generate poor immunological response in immunocompromised (IC) patients (1–6), including solid organ transplant (SOT), an autoimmune (AI) disorder, blood cancer, and chronic inflammatory diseases (CIDs) patients, etc. (~7 million US adults) (7–11).

All mRNA therapeutics must undergo endosomal escape following their uptake by the cells and get translated (protein synthesis) into encoded target antigen (Ag) protein by ribosomes in the cytosol. Then, the immunogenic epitopes are presented to T and B cells to stimulate the immune system and produce cellular and antibody (Ab) responses. The translation process is a key preceding step to mRNA vaccine-induced generation of immune response. Thus, modulation of translation process is expected to influence COVID mRNA vaccine effectiveness (VE). Although the current Co-mV is sequence optimized to increase mRNA stability and maximize protein translation, several determinants such as nutrient availability, genetics, cellular stress, ribosome quality, inclusion of modified nucleotides, mRNA secondary structure, and importantly, drugs/inhibitors present in the host system, can interfere with the process of translation and its kinetics. Among different types of inhibitors/drugs, certain immunosuppressants (ISs) such as glucocorticoids, tumor necrosis factor inhibitors, the combination of inhibitors of tacrolimus (T) (calcineurin inhibitor), mycophenolate (M) (antimetabolite), mammalian target of rapamycin (mTORi), including rapamycin/sirolimus (S), and prednisone (P) (glucocorticoids) (TMP and TSP combinations) that are commonly used to achieve and maintain disease response and remission in IC patients including SOT patients, have been individually or in combination (TMP/TSP) reported involve in translation process (protein synthesis) directly or indirectly by modulating the related physiological processes (7–22).

However, till date there is no evidence to determine empirically whether there is any interaction between the ISs and Co-mV translation process. Therefore, such studies are warranted to enable the scientific community to define a mechanistic basis for dampened effectiveness of Co-mV in IC setting and also help with choosing the right combination of drugs to use for certain time interval during and after mRNA vaccination to minimize the impact on mRNA translation process. Here, using clinical evidence in tandem with proof-of-principle based *in vitro* and *in vivo* animal experiments, we assess whether the COVID-19 mRNA VE is altered through impaired Sp antigen translation in IC SOT patients that are on immunosuppressive medications. Further, we have evaluated a few simple and practical approaches to improve Co-mV translation under immunocompromised setting.

## Materials and methods

### Patient samples

The 2D Co-mV experiments in this study included a total of 309 subjects vaccinated for SARS-CoV-2 with 2D of Pfizer-BioNTech (New York, NY, USA) or Moderna COVID-19 mRNA vaccines (Cambridge, MA, USA), with immunocompromised group consisting of SOT recipients, neuroimmunology, and various cancer subjects on ISs (n=259), and naïve (non-transplanted and non-exposed to COVID-19) group not on any prescribed immunosuppressant (n=50). The 3D COVID-19 vaccine experiment (of mRNA vaccines and Regeneron antibody) included a total of 910 individuals with 67 SOT recipients who received 3D vaccines and compared them with 843 non-transplanted naïve individuals. This study excluded prior COVID-19 cases. The institutional review board of the University of Texas Southwestern Medical Center approved this study.

Antibody responses were semi-quantitatively assessed using serum samples on the Alinity i platform (Abbott Laboratories) with the FDA-approved SARS-CoV-2 anti-nucleocapsid protein IgG assay (IgG_NC_), SARS-CoV-2 anti-spike protein IgM assay (IgM_Sp_), or SARS-CoV-2 anti-spike protein IgG II assay (IgG_Sp_), as described (23). Index values of ≥1.4 (IgG_NC_), ≥1.0 (IgM_Sp_), and ≥50 AU/mL (IgG_Sp_) were interpreted as positive per the manufacturer’s recommended threshold. IgG_NC_ positivity informs natural SARS-CoV-2 infection, while IgG_Sp_/IgM_Sp_ positivity strongly correlates with the emergence of natural or vaccine-driven neutralizing immunity (1, 23).

### Non-clinical proof-of-concept experiments

These non-clinical non-proof-of-concept experiments were performed *in vitro* using HEK293 cells or using Balb/C mice in strict accordance with the *Guide for Care and Use of Laboratory Animals* approved by the UT Southwestern Institutional Animal Care and Use Committee (IACUC).

### *In vitro* experiments

*In vitro* assays were performed in HEK-293 cells cultured in Dulbecco’s Modified Eagle’s Medium (DMEM) with penicillin/streptomycin, L-glutamine and 10% fetal bovine serum (FBS) and maintained at 37°C in a humidified atmosphere with 5% CO_2_.

### Doses of Co-mV and different drugs used *in vitro* experiments

The dose and time for the *in vitro* Co-mV expression experiments were based on the first SARS-CoV-2 mRNA vaccine design and validation studies (24). We used HEK293 cells as the *in vitro* cellular model because of its amenability to transfection and transduction using a variety of chemical and physical method and thereof on a wide application for gene manipulation experiments *in vitro*. To mimic the clinical setting, we used the therapeutically equivalent doses of commonly used IS drugs as previously described (25–28). When individually used, the concentrations of each of the drugs were as follows: T (25 ng/mL), S (25 ng/mL), M (10 μM), and P (10 μM) and when used in combinations the concentrations of each of the drugs were T (12.5 ng/mL), S (12.5 ng/mL), M (4 μM), and P (4 μM).

### Cell transfection

For the experiments involving transfection, 10^6^ HEK293 cells cultured in DMEM media were seeded in 6-well plates and grown overnight to obtain 70% confluency. The cells were pretreated with the drugs for 16 h before transfection. 1-3 µg of Pfizer-BioNTech Co-mV was appropriately diluted in serum free OPTI-MEM I media, incubated at room temperature (RT) for 5 mins, and added to the plates. 1 h post-transfection, DMEM media containing 20% FBS was added, and the plates were returned to incubator for additional 24 h to determine the effect of ISs on the Co-mV-induced expression of spike protein and translation process’ surrogates.

### Immunoblotting

Following experimental treatments, HEK293 cells were gently washed with ice-cold phosphate buffered saline (PBS) and lysed in ice-cold RIPA buffer with cOmplete™ Mini Protease Inhibitor Cocktail (Roche Diagnostics) and PhosSTOP (Roche Diagnostics). Cells were scraped and transferred into a 1.5ml Eppendorf tube and incubated on ice for 10 minutes and vortexed. The lysates were centrifuged at 13,000rpm for 5 mins at 4°C and supernatants were collected. An equal amount of protein (10 μg) from different treatment samples were separated by SDS-PAGE gel with 100V for 1.5 h and transferred onto nitrocellulose membrane. The membranes were then blocked with 5% non-fat milk in TBST for 1h at RT and probed against specific primary antibody for SARS-CoV-2 spike (GeneTex, Cat# GTX632604), S6 ribosomal protein (Cell Signaling, Cat#2217), phospho-S6 ribosomal protein (Cell Signaling, Cat# 2211), and β-actin (Abcam, Cat# ab49900) overnight in 4°C. After washing, the membranes were incubated for 1h with corresponding horseradish peroxidase (HRP)-conjugated secondary antibody. Washed blots were immunodetected using Pierce™ ECL Western Blotting Substrate (ThermoFisher, Cat# 32106). β-actin expression was used to normalize loading. The immunoreactive signals were quantified by densitometry using Image J software.

### RNA extraction and real-time quantitative RT-PCR

Total cellular RNA was extracted using RNeasy Kit (Qiagen, Valencia, CA). 1 µg of RNA reverse transcribed to cDNA using Bio-Rad iScript reagents according to instructions (Bio-Rad Laboratories, Cat# 1708890).

Quantitative real-time RT-PCR was performed using 1/10^th^ of cDNA prepared as above together with a set of 4 different primer sets of interest as below spanning the spike region of SARS-CoV-2 mRNA sequences found in the Co-mV individually in a 10 µl SYBR green Supermix (Bio-Rad Laboratories, Cat# 1725270) and amplified using CFX384 Touch Real-Time PCR Detection System (Bio-Rad Laboratories). The thermal cycling conditions used include 50°C/2 min; 95°C/10 min followed by 40 PCR cycles at 95°C/15 sec and 60°C/1 min. Relative expression was quantified using Ct values, and expression fold-change was calculated by normalization to the Ct of the housekeeping gene, β-actin, according to the 2^-ΔΔCt^ methods (29, 30).

**Table:** 

**Primer #**	**Forward 5’-3’**	**Reverse 5’-3’**
1	TTCAGCAACGTGACCTGGTT	TTGTTCACGATCAGCAGGCT
2	TGCGAGTTCCAGTTCTGCAA	GATAGGGGTGTGCTTGCTGT
3	GAGCCCCAGATCATCACCAC	CAGATTCTTGGCCACCTCGT
4	ATGTTCGTGTTCCTGGTGCT	AACCAGGTCACGTTGCTGAA

### *In vivo* experiments

Array of *in vivo* non-clinical proof-of-concept experiments involved 8-10 weeks old healthy Balb/c (Envigo) female mice. All mice were housed in pathogen-free conditions were acclimatized for 1 week before the start of the experiment. Drug treatment was started 7 days before Co-mV treatment. Drugs were administered by oral gavage daily and mice were assigned randomly by body weight to Group-1 – PBS; and Group-2 - Tacrolimus (12.5mg/kg) + Sirolimus (12.5mg/kg) + Prednisone (5mg/kg). Priming dose of 2 μg of Pfizer-BioNTech Co-mV in 50 µl of PBS was intramuscularly injected on the left thigh of mice that was separated by 21-days with a second (booster) dose of the same amount of vaccine. Body weight was measured every 7 days and 0.5ml of blood collected from each mouse in EDTA-coated tube was processed for serum separation, which was used in downstream spike antigen analysis and viral antibody (IgG and IgM) profiling.

### Justification of Co-mV and different drugs used *in vivo* Balb/c experiments

The choice and relevance of the mouse and dose of the Co-mV used in non-clinical proof-of-concept experiments was based on the Comirnaty assessment report and pre-clinical studies followed for RNA-based vaccine development (24, 31). The concentration of the drugs used *in vivo* was based on the previously published pre-clinical studies and these have been extensively used in mice over a wide range of doses (32–37).

### Sandwich ELISA to assess the SARS-CoV-2 Spike immunogen

The levels of SARS-CoV-2 viral Sp immunogen in the serum samples from the immunized mice with and without TSP treatments were determined using SARS-CoV-2 Sp RBD sandwich ELISA kit per manufacturer’s instructions (GeneTex, GTX536267). Briefly, 50 μl of each standard or 1:10 diluted sample was placed into appropriate wells coated with mouse anti-Sp monoclonal antibody and incubated at RT for 2h. After aspiration and 6 washes with wash buffer, 50 μl of 1x conjugate solution containing horseradish peroxidase-conjugated rabbit monoclonal antibody was added and incubated at RT for 1h. Followed by another 6 washes, tetramethylbenzidine (TMB) substrate solution (100 μl) was added, and incubated in darkness for 15 mins at RT. The reaction was terminated by adding 100 μl of stop solution containing 1N sulfuric acid, and the absorbance at 450 nm (A450) was determined within 15 mins. The concentration was calculated from the standards and expressed as pg/mL according to kit instructions.

### Profiling of antibodies to viral antigens using microarrays

This study used the detection of antibodies to 42 different viral antigens, including SARS-CoV-2 using a custom developed and a highly sensitive fluorescent-based multiplex microarray assay at our Microarray and Immune Phenotyping core facility of UT Southwestern Medical Center. The antibody profiling was carried out as described by us previously (38, 39). Serially diluted mouse IgG and IgM were added as internal controls. Briefly, DNAse-I-pretreated 2 µl serum samples from mice were diluted 1:50 in PBST buffer were incubated in duplicates in the viral antigen nitrocellulose film slides/arrays (Grace Bio-Labs) printed with the viral antigens and control proteins. The outcome of serum/plasma-derived mouse antibodies binding with arrayed antigens into a readout were detected using a Genepix 4200A scanner (Molecular Device) with laser wavelength of 532 nm and 635 nm, after probing with cy3-conjugated anti-mouse IgG (1:2000, Jackson ImmunoResearch Laboratories) and cy5-conjugated anti-mouse IgM (1:2000, Jackson ImmunoResearch Laboratories). The resulting images were analyzed using Genepix Pro 7.0 software (Molecular Devices). The averaged fluorescent signal intensity of each antigen was subtracted by signal from the spot background and the PBS control and normalized to mouse IgG or IgM (internal controls) to obtain the normalized fluorescence intensity (NFI). This served as a quantitative measurement of the binding capacity of each mouse sample-derived antibodies with the corresponding viral antigen analytes. The NFI of each analyte was used to generate heatmaps using Graphpad Prism 10.1 software.

### Statistical analysis

Statistical differences were determined using one-way or two-way ANOVA when experiments involved more than two groups. Student’s t-test was used for experiments involving only two groups. The analysis was carried out using GraphPad software 9.3.1 and p<0.05 was considered as statistically significant for the *in vitro* and *in vivo* experiments.

## Results

### Demographics of the IC patients and comparison of COVID-19 vaccine responses in ICs vs healthy controls

The clinical data in 2D Co-mV experiments included a total of 259 IC patients and 50 controls meeting the inclusion criteria of no prior COVID-19. The IC group was found to be older than the control group (mean ± SD; 60 ± 15 vs 52 ± 13 years) and more likely to be males (54% vs 44%) (**Table 1**). More than one-quarter (27%) IC subjects had been subjected to either treatment with S or M combinations of ISs (**Figure S1**). In the 3D group, the non-transplanted controls (NT) had 843 subjects with a mean age 65 ± 14 years and the SOT group had 67 patients with a mean age 55 ± 15 years (**Table 1**). Male subjects were more common among the SOT group when compared to NT (68.7% vs 46.6%) (**Table 1**). Irrespective of 2D or 3D and controls or IC group, our study population had a greater number of patients vaccinated with Pfizer formulation relative to Moderna vaccines (**Table 1**).

**Table 1:** Demographic and clinical characteristics of study participants.

Information	2D Co-mV		3D Co-mV	
	HC (n=50) (%)	IC (n=259) (%)	NT (n=843) (%)	SOT (n=67) (%)
**Age (Years)**				
<19	–	–	1 (0.1)	–
20-39	9 (18)	32 (12)	60 (7.1)	9 (13.4)
40-59	26 (52)	72 (28)	187 (22.2)	29 (43.3)
>60	15 (30)	155 (60)	595 (70.6)	29 (43.3)
**Sex**				
Female	28 (56)	119 (46)	449 (53.3)	21 (31.3)
Male	22 (44)	140 (54)	393 (46.6)	46 (68.7)
Declined/Unknown	–	–	1 (0.1)	–
**Race**				
Non-white	27 (54)	45 (17)	87 (10.3)	22 (33)
White	20 (40)	202 (78)	670 (79.5)	41 (61)
Declined/Unknown	3 (6)	12 (5)	86 (10.2)	4 (6)
**Vaccine**				
BNT162b2 (*Pfizer-BioNTech*)	47 (94)	195 (75)	554 (65.7)	52 (78)
mRNA-1273 (*Moderna*)	3 (6)	64 (25)	287 (34.1)	15 (22)
Unknown	–	–	2 (0.2)	–

After immunizing with 2D and 3D Co-mV, 107 (41%) and 10 (15%) IC patients had IgG_Sp_ levels less than the manufacturer-recommended positivity threshold of 50 AU/mL. While 0 (0%) and 54 (6%) control subjects (HC & NT) had IgG_Sp_ levels less than 50 AU/mL in the 2D and 3D Co-mV format experiments (**Figure S2** and **Table 2A**). In particular, previous reports have shown that IgG_Sp_ antibody titers of ≥ 4160 AU/mL correspond to a neutralizing titer (40, 41) and here, we have found that 1 in 6 IC patients (16%) was able to generate neutralizing titers after 2 Co-mV doses compared to healthy controls (64%) (**Table 2B**). Although, the 3^rd^ dose improved the IgG response over neutralizing titer of 4160 AU/mL, despite 3 Co-mV doses, still over half the population (57%) in the immunocompromised SOT group had IgG response below the neutralizing titers (**Table 2B**). No significant Co-mV-associated adverse effects were noted in the IC patients receiving 2 or more doses.

**Table 2A:** Estimation of 2D and 3D COVID-19 vaccine administered IC cohorts exhibiting IgG titer above manufacturer recommended threshold of 50 AU/mL.

Dose of COVID-19 Vaccine		No. of patients with IgG above manufacturer threshold	Total No. of patients	% of patients with IgG above manufacturer threshold
2D Co-mV	HC	50	50	100
	IC	152	259	59
3D Co-mV	NT	789	843	94
	SOT	57	67	85

**Table 2B:** Estimation of 2D and 3D COVID-19 vaccine administered IC cohorts exhibiting IgG titer above a neutralizing threshold of 4160 AU/mL (41).

Dose of COVID-19 Vaccine		No. of patients with IgG above a neutralizing threshold	Total No. of patients	% of patients with IgG above a neutralizing threshold
2D Co-mV	HC	32	50	64.0
	IC	43	259	16.2
3D Co-mV	NT	508	843	60.3
	SOT	29	67	43.3

### The muted Co-mV-induced antibody response in IC cohorts was further restrained by mycophenolate and sirolimus treatment

**Figure 1:**
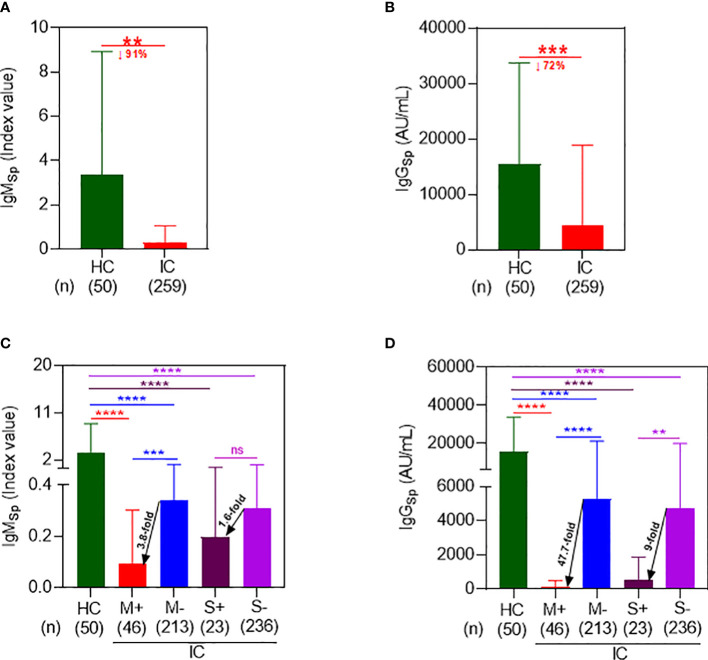
Immunocompromised patients on mycophenolate and sirolimus evoked a poor spike antibody response following 2D Co-mV administration. **(A)** Changes in IgM_Sp_ antibody (index value) levels following administration of 2D of Co-mV administration in HC subjects and IC patients receiving routine ISs combination that included mycophenolate and sirolimus. **(B)** Comparison of IgG_Sp_ (AU/mL) levels in 2D Co-mV-administered IC patients maintained on routine ISs combination that included mycophenolate and sirolimus with immunocompetent HC subjects. **(C)** IgM_Sp_ antibody concentrations among HC and different IC patient groups categorized based on the presence and absence of mycophenolate and sirolimus on their ISs regimen. **(D)** Quantification of IgG_Sp_ in HC subjects and IC participants receiving or not receiving mycophenolate and sirolimus as part of their immunomodulatory therapy after 2D Co-mV immunization. In panels **(A–D)**, the box plot indicates mean ± SD. For the panels **(A, B)**, comparison of the means among the groups and the statistical difference was established using two-tailed Welch’s t-test and for panels **(C, D)**, using Kruskal-Wallis One-Way ANOVA. In panels **(A–D)**, the significant interaction denotes the following: (** p = 0.0003; *** p = 0.0001; **** p < 0.0001 *vs* the respective compared group, as indicated). 2D, two doses; Co-mV, COVID-19 mRNA vaccines; HC, healthy controls; IC, immunocompromised; ISs, immunosuppressants; Sp, spike; IgM, immunoglobulin M; IgG, immunoglobulin G; AU, arbitrary units; SD, standard deviation; ANOVA, analysis of variance; ↓, decrease; M+, receiving mycophenolate along with other ISs; M-, not receiving mycophenolate alone; S+, receiving sirolimus along with other ISs; S-, not receiving sirolimus alone.

Following a 2-dose regimen of Co-mV, the HC displayed a mean Sp IgM index value of 3.3 ± 5.6 which was found to be significantly attenuated in IC patients by 91% with a mean index value of 0.3 ± 0.8 (p=0.0004; **Figure 1A**). In parallel to IgM, the IC patients after 2-doses of Co-mV showed a significant diminution in mean Sp IgG levels by 72% with a mean value of 4365 ± 14523 AU/mL relative to HC group with a mean level of 15454 ± 18289 AU/mL (p=0.0002; **Figure 1B**). This result clearly shows that IC patients that were on ISs had poor IgM and IgG antibody response to Co-mV.

Next, to determine the effect of M and S on the Co-mV-evoked IgM, we segregated and analyzed the IC cohorts based on their particular ISs treatment with M or S. 2D Co-mV-administered IC patients who were on M treatment showed a significant reduction in the mean Sp IgM levels of 0.09 ± 0.21 (**Figure 1C**; lane 1 vs 2; 37-fold; p<0.0001) when compared with 2D vaccinated non-transplanted healthy subjects that had a mean IgM index value of 3.3 ± 5.6. This decline was less pronounced by only 9.7-fold in the IC patients that were not on M (0.34 ± 0.83 vs 3.3 ± 5.6 index value; lane 3 vs 1; p<0.0001). Interestingly, among the 2D immunized IC patients, those that were on M showed a 3.8-fold reduction in mean IgM levels relative to the group that was not on M (0.09 ± 0.21 vs 0.34 ± 0.83 index value; lane 2 vs 3; p=0.0002). Similarly, S treatment significantly dampened the Co-mV-induced mean Sp IgM levels in IC patients to 0.2 ± 0.36 index value when compared to vaccinated healthy subjects by 17-fold (lane 4 vs 1; p<0.0001). This decrease was less pronounced by 10.6-fold in vaccinated IC patients that were not on S (0.31 ± 0.79 vs 3.3 ± 5.6 index value; lane 5 vs 1; p<0.0001). Among the immunized IC patients, S treatment abridged the mean IgM index value by 1.6-fold relative to the group that was not on S (0.2vs 0.31 index value; lane 4 vs 5; p>0.9999). This data indicates that M and S, a class of immunosuppressants used to treat transplant and autoimmune patients, can inhibit the initial production of IgM (the first antibody to develop as part of humoral immune response), which mainly requires translation of spike antigen to generate an immune response besides other processes such as antigen processing, presentation, and recognition.

Similarly, 2D Co-mV administered IC patients that were on M treatment showed a significant attenuation of Co-mV-evoked Sp IgG levels by 139-fold with a mean of 110.8 ± 390.1 AU/mL relative to 2-dose Co-mV-induced Sp IgG mean levels, 15454 ± 18289 in non-transplanted healthy subjects (**Figure 1D**; lane 2 vs 1; p<0.0001). The extent of this diminution was found to be less pronounced by only 3-fold in 2D-vaccinated IC patients that were not on M (5284 ± 15871 vs 15454 ± 18289; lane 3 vs 1; p<0.0001). Further, among the vaccinated IC patients, those that were on M showed a 47.7-fold repression in mean IgG levels relative to the group that was not on M (**Figure 1D**; 110.8 vs 5284 AU/mL; lane 2 vs 3; p<0.0001). Similarly, S treatment significantly dampened the 2-dose Co-mV-induced mean Sp IgG levels by 29-fold in IC patients when compared to Co-mV-administered non-transplanted healthy subjects (**Figure 1D**; 528.5 ± 1334 vs 15454 ± 18289 AU/mL; lane 4 vs 1; p<0.0001). The magnitude of this inhibition was less pronounced by 3.3-fold in IC patients that were not on S (4739 ± 15160 vs 15454 ± 18289; lane 5 vs 1; p<0.0001). Among the 2D-immunized IC patients, those that were on S showed a 9-fold reduction in mean IgG levels relative to the group that was not on S (**Figure 1D**; 528.5 vs 4739; lane 5 vs 4; p=0.0021). This data clearly indicates that mycophenolate and sirolimus treatment in IC patients led to significant repression of the Co-mV-induced immune response based on IgG levels, a process that is also dependent on translation of Spike antigen.

### Comparison of Sp antigen to Sp antibody levels in IC patients based binned Ag thresholds

To demonstrate the direct production of Sp protein *via* translation after Co-mV, we measured Sp immunogen levels and compared it with Sp antibody levels in the same patients. Due to physiological differences, it is well known that different individuals develop different thresholds of Ag and associated antibody response. In our study subjects, we observed a wide range of Sp Ag expression from 15.8 to 11,366.47 pg/mL. Using an arbitrary cut-off of 200 pg/mL for Sp Ag levels based on ~≤5% from the highest Ag value found in the HC group (4025 pg/mL), we could bin subjects into low (≤ 200 pg/mL; Lo-bin) and high (>200 pg/mL; Hi-bin) groups. This enabled us to tease out the differences in the Ab and Ab levels in HC and IC patients.

**Figure 2:**
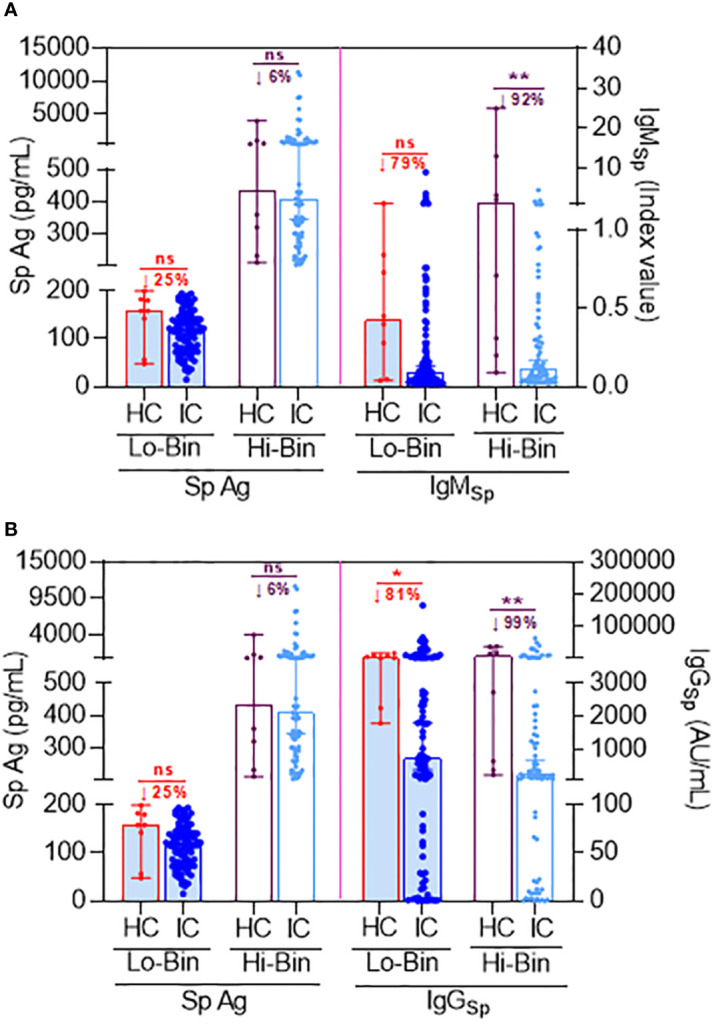
Comparison of Sp antigen to Sp antibody levels following 2D Co-mV administration under binned Ag thresholds between HC and IC patients that are on ISs. **(A)** Figure representing the effect of 2D Co-mV administration on Sp Ag levels (left pane) and IgM_Sp_ antibody levels (right pane) created using bin algorithm by exploiting specific threshold rules of Sp Ag (as described in results section) in corresponding HC subjects and IC patients receiving routine ISs combination that included mycophenolate and sirolimus. **(B)** Figure representing the effect of 2D Co-mV administration on Sp Ag levels (left pane) and IgG_Sp_ antibody levels (right pane) created using bin algorithm by exploiting specific threshold rules of Sp Ag (as described in results section) in corresponding HC subjects and IC patients receiving routine ISs combination that included mycophenolate and sirolimus. Box plot represents median with 95% CI. Comparison of the medians among the groups and the statistical difference was established using a two-tailed Mann Whitney t-test. The significant interaction denotes the following: (* p = 0.0144; ** p = 0.0013 **(A)** and 0.0089 **(B)**; ns = p not significant *vs* the respective compared group, as indicated). 2D, two doses; Co-mV, COVID-19 mRNA vaccines; HC, healthy controls; IC, immunocompromised; ISs, immunosuppressants; Sp, spike; IgM, immunoglobulin M; IgG, immunoglobulin G; AU, arbitrary units; CI, confidence interval; Ag; antigen; Lo, low; Hi, high; ↓, decrease.

In HC, the median Sp Ag level in the Lo and Hi bin group was found to be 158.2 pg/mL (99% CI, 48.2 to 199.7 pg/mL) and 434.7 pg/mL (99% CI, 209 to 4025 pg/mL), respectively (lane 1 & 3; **Figure 2A**). While in IC, the median Sp Ag level in the Lo and Hi basket was found to be 119.2 pg/mL (96% CI, 107.9 to 131.4 pg/mL) and 409.2 pg/mL (96% CI, 344.3 to 530.1 pg/mL), respectively (lane 2 & lane 4; **Figure 2A**). This was about a 25% and 6% reduction in Lo and Hi bin group compared to respective baskets in HC.

Since the first antibodies to be produced in a humoral immune response following an exposure to an Ag are always IgM, we first assessed the status of Sp Ag with respect to IgM production in the same patients. The IgM production was lowered by 79% in low-bin IC (≤200 pg/mL Sp Ag) relative to Lo-bin of HC (lane 6 vs 5; **Figure 2A**). Likewise, there was 92% reduction of IgM in Hi-bin IC group relative to the Hi-bin HC (>201 pg/mL Sp Ag) (lane 8 vs 7; **Figure 2A**). Similarly, it was found that the IgG production was lowered by 81% in Lo-bin IC (≤200 pg/mL spike Ag) relative to Lo-bin HC (lane 6 vs 5; **Figure 2B**). This was found to be 99% reduced in Hi-bin IC group (>201 pg/mL spike Ag). Overall, these results are suggestive that IC who are on ISs show a restrained Sp antibody (IgM and IgG) production in response to Co-mV, which is at least partially preceded by an impairment in Sp Ag generation.

### *In vitro* assessment of translation of target Ag (Sp) following Co-mV expression

The ISs such as T, S, M, and P and their widely applied combination of TMP/TSP used in immunosuppressed populations can either directly or indirectly perturb mRNA translation (17–22, 42). However, no data exists whether these ISs either individually or in combination repress translation of Co-mV, which could be one of the potential reasons for the dampened Co-mV response seen in IC patients. To address this question, we assessed the direct production of Sp protein generated as a result of translation following Co-mV expression in HEK293 cells with and without the presence of indicated drugs.

First, the optimization experiments clearly indicated that Co-mV treatment at different concentrations can produce Sp protein *in vitro* for 24 h, as detected by immunoblotting (**Figure S3**). Importantly, this expression appeared to be optimal with 2 μg dose and not requiring any external transfection agent, like lipofectamine (**Figure S3A**). The time points 1, 3, and 6 h were not sufficient for the Co-mV expression (**Figure S3B**). Thus, all the subsequent *in vitro* experiments just used the addition of 2 μg of Co-mV for at least 24 h without any addition of transfection agents to the cells.

### Impact of ISs on the translational capacity of target immunogen (Sp) following Co-mV expression

**Figure 3:**
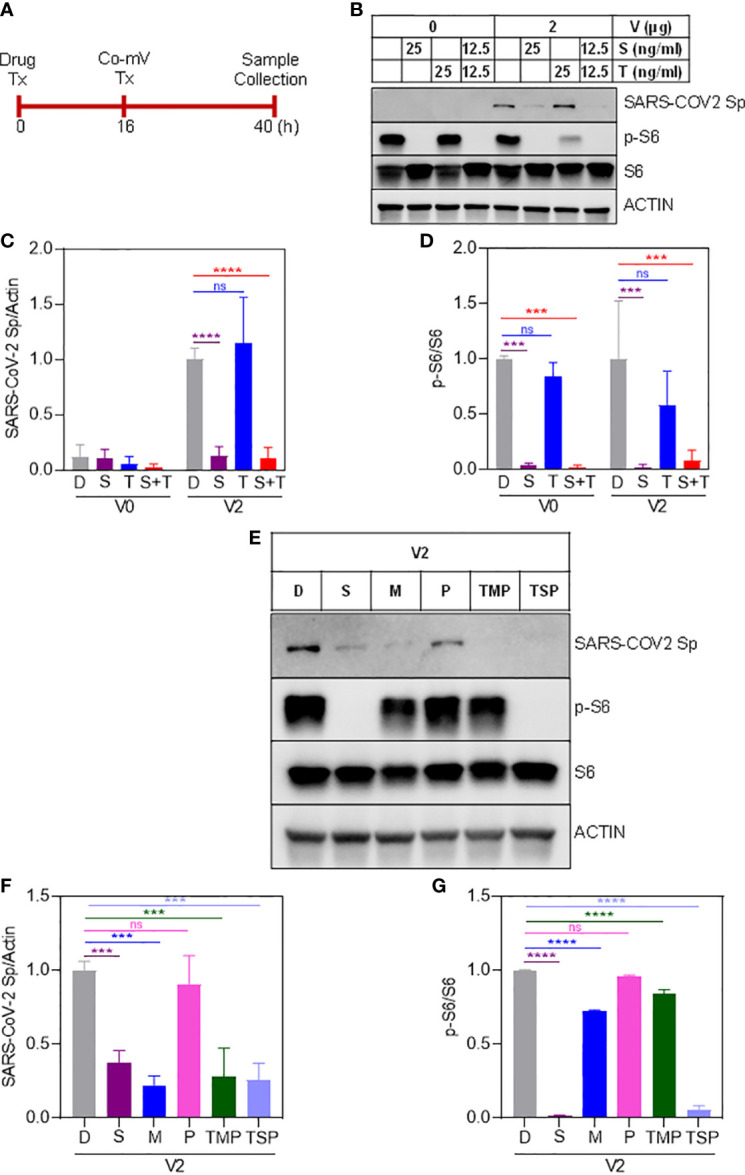
Sirolimus and mycophenolate reduces Pfizer Co-mV-induced expression of Sp protein and phosphorylation of pS6 in HEK293 cells. **(A)** Drug and 2 μg Pfizer BioNTech Co-mV treatment schedule in HEK293 cells. **(B)** Western blot for SARS-CoV-2 Sp protein expression in HEK293 cells pre-treated over 16 h with 25 ng/mL each individually with sirolimus and tacrolimus (FK506), or their combination at 12.5 ng/mL each and with an additional 24 h treatment along with 2 μg Pfizer BioNTech Co-mV [as described in panel **(A)**]. DMSO treatment served as controls. The same blots were stripped and reprobed with pS6, S6, and β-actin antibodies. **(C)** Expression of SARS-CoV-2 Sp protein normalized to actin and **(D)** phospho-specific protein S6 to total S6 in the immunoblots illustrated in panel **(B)** were quantitated by densitometry and the relative levels to DMSO controls were represented. **(E)** HEK293 cells were pre-treatment for 16 h with the indicated drugs either alone or in a combination of TSP or TMP at the concentrations (described in materials and methods) and with an additional 24 h treatment along with 2 μg Pfizer BioNTech Co-mV. At the end of the experimental period, equal amount of protein lysates from all the groups were analyzed using Western blotting for protein expression of SARS-CoV-2 Sp, phospho-specific pS6, total S6, and actin as a loading control. **(F, G)** Quantification of the immunoblots in panel **(E)** expressed as a ratio of SARS-CoV-2 Sp to Actin; and phospho-specific protein S6 to total S6, respectively. Relative values to DMSO controls were shown. Data was expressed as the mean ± SD (n=3). For panels **(C, D)** that involves 2 different doses, the statistical inferences between DMSO and treatment groups were made from a two-way ANOVA, Tukey *post-hoc* multiple comparisons and for panels (In panel **(C)**, **** p < 0.0001; In panel **(D)**, *** p = 0.0004 [V0 – D vs S]; *** p = 0.0003 [V0 - D vs S+T]; *** p = 0.0003 [V2 – D vs S]; *** p = 0.0006 [V2 – D vs S+T]). For panels **(F, G)** that involves a single dose, the statistical inferences were made from a one-way ANOVA with Tukey *post hoc* multiple comparisons test (In panel **(F)**, *** p = 0.0008 [D vs S]; *** p = 0.0001 [D vs M]; *** p = 0.0002 [D vs TMP & D vs TSP]; ns – not significant; In panel **(G)**, p = **** p < 0.0001, ns – not significant). Co-mV, COVID-19 mRNA vaccine; Tx, treatment; V0, no CO-mV; V2, 2 μg Co-mV; D or C, dimethyl sulfoxide (DMSO); S, sirolimus; T, tacrolimus; P, prednisone; SARS-CoV-2 Sp, severe acute respiratory syndrome coronavirus 2 spike; S6, S6 ribosomal protein; pS6, phospho-S6 ribosomal protein.

Next, we sought to determine whether commonly used ISs either individually and/or in combination reduce the protein expression of Sp immunogen that was generated as a result of translation of the transfected Co-mV (**Figure 3A**). The immunoblotting results indicated that the drugs S, M, but not T and P significantly reduced the Co-mV-induced Sp immunogen expression compared to the no-drug control (**Figures 3B, C, E, F**). In addition, the commonly used combo TMP and TSP also resulted in a significant reduction of the Co-mV-induced Sp immunogen expression (**Figures 3E, F**). Importantly, this was consistent with significant inhibition of the phosphorylation status of p-S6 that was widely used as translation process surrogates (p<0.05; **Figures 3E, G**). M-induced p-S6 inhibition was relatively less pronounced than sirolimus. While the total levels of S6 remained unaltered (**Figures 3B, D, E, G**). These results suggest that some of the commonly used ISs and their TSP and TMP combination can inhibit the Sp protein translation and likely account for the impaired vaccine response that is normally observed in at-risk IC conditions.

### ISs-induced diminution of Sp immunogen levels following Co-mV treatment is not due to restrained mRNA intake

**Figure 4:**
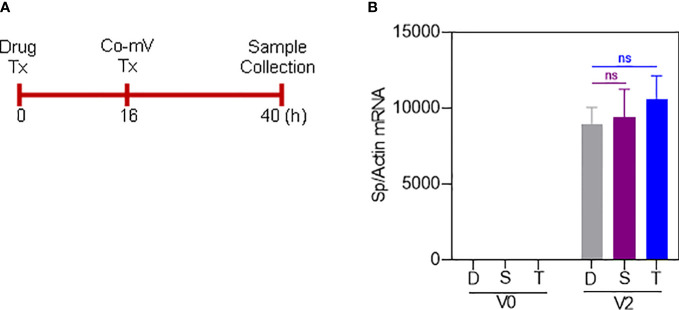
Sirolimus and Tacrolimus did not alter the uptake of Pfizer-BioNTech Co-mV in HEK293 cells. **(A)** Schematic of the drug and Pfizer BioNTech Co-mV treatment schedule in HEK293 cells. **(B)** HEK293 cells were transfected with 2 μg Pfizer Co-mV and without lipofectamine. RNA was purified and real-time qRT-PCR was performed using primer set 1 targeting a spike region in Pfizer-BioNTech Co-mV (as provided in materials and methods). RNA levels of spike were presented as 2^−ΔΔCT^ values relative to house-keeping gene, actin. Results were expressed as the mean ± SD from four independent experiments (n = 4). Differences between the non-transfected and transfected groups and with DMSO and individual drug treatments were analyzed using a two-way ANOVA, Tukey *post-hoc* tests (multiple comparisons) (ns – not significant vs DMSO treated controls, as indicated). Tx, treatment; Co-mV, COVID-19 mRNA vaccine; V0, no CO-mV; V2, 2 μg Co-mV; D, dimethyl sulfoxide (DMSO); S, sirolimus; T, tacrolimus; Sp, spike; qRT-PCR, real-time quantitative reverse transcription-polymerase chain reaction; SD, standard deviation; CT, cycle threshold; 2^−ΔΔCT^, 2ˆ(–delta delta CT), a comparative CT method quantification.

The ISs can also be argued to interfere with the diminished uptake of mRNA, thereby leading to a lack of adequate levels of Sp immunogen following Co-mV treatment. To address this, we utilized 4 primer sets spanning SARS-CoV-2 region used in the Co-mV and determined their comparable levels of Sp mRNA expressions in the drug and no-drug pre-treated Co-mV transfected groups (**Figure 4A**). After Co-mV treatments, we observed that the comparable levels of different Sp mRNA transcripts using these 4 primer sets could be detected (**Figure S4**) indicating the feasibility of testing the Co-mV uptake. Choosing primer set 1, we next detected that there was no change in the comparable levels of Sp mRNA between the IS drugs and no-drug control (**Figure 4B**). This indicates that COVID mRNA vaccine entered cells regardless of IS treatment and, thus, the ISs-induced mRNA uptake may perhaps not be a limiting factor for the observed inadequate levels of Sp immunogen following Co-mV treatment.

### TSP combo treatment attenuated Co-mV-induced IgM and IgG antibodies against SARS-CoV-2 S-protein *in vivo*

To-date there is no experimental evidence showing whether the ISs used in organ transplant recipients can affect both the Co-mV-induced antigen and antibody reactivities. Thus, to address the effect of TSP, a commonly used ISs combo on Co-mV-evoked immunogenicity, we first utilized a viral antibody profiling-based microarray and assessed the IgM and IgG reactivities against different SARS-CoV-2 protein spanning the whole antigen S or for any specific domain such as RNA binding domain (RBD), S1, & S2 domains. This *in vivo* experiment compared sera from vehicle-treated Co-mV-immunized mice with TSP combo-treated Co-mV-immunized group. No detrimental local trauma (data not shown) or body weight changes were seen in mice following Co-mV immunization at the indicated periods of time (**Figure S5**). Further, there were no apparent signs of infection, inflammation, bleeding disorder, or mortality associated with Co-mV administration (data not shown).

**Figure 5:**
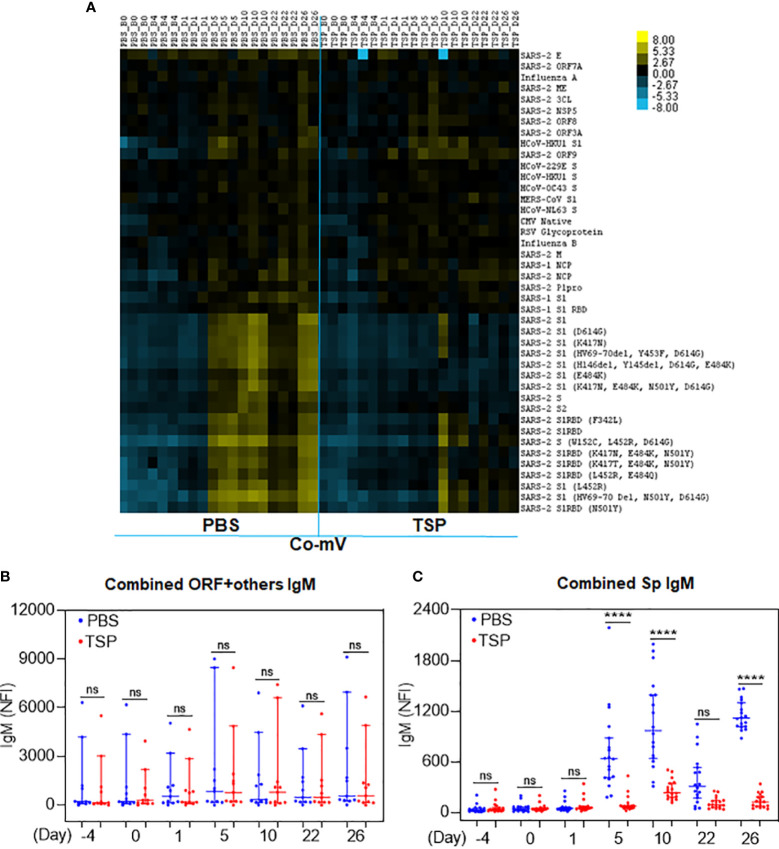
TSP drug combination attenuated the Co-mV- production of IgM antibodies against SARS-CoV-2 viral antigens in Balb/C mice *in vivo*. IgM antibody profile to SARS-CoV-2 viral antigens in the PBS vehicle treated and TSP drug combination treated Balb/C mice that were immunized with Pfizer Co-mV, as indicated in materials and methods. **(A)** The heatmap shows IgM reactivity expressed in terms of row z-score for a respective antigen across different mice samples. Each antigen was organized into rows with serum specimens organized into columns classified as B0 (Baseline 0^th^ day, 6 samples), B4 (Baseline 4^th^ day – 6 samples), D1 (day 1 after PBS or TSP administration – 3 samples each), D5 (day 5 after PBS or TSP administration – 3 samples each), D10 (day 10 after PBS or TSP treatment – 3 samples each), D22 (day 22 post PBS or TSP treatment – 3 samples each), D26 (day 26 after PBS or TSP treatment – 2 samples each). Reactivity was represented by color (Light Blue = low, Black = mid, Yellow = high). The heatmap has normalized row z-score values, a typical scaling method that helps better visualization of analytes with varying trends in the expression/reactivity between samples. While a normalized row z-score can better represent the non-randomness of directionality within a dataset, a negative z-score does not indicate a complete absence of expression/reactivity. A negative z-score means comparatively a lower raw scores/absolute expression. **(B)** Analysis of panel A’s heatmap data illustrating the sum of IgM NFI responses for combination of SARS-CoV-2 antigens representing different domains devoid of spike such as 3C-like protease (SARS-2 3CL), envelope (SARS-2 E), nucleocapsid (SARS-2 NCP), M protein (SARS-2 M), non-structural protein 5 from ORF1 (SARS-2 NSP5), open reading frames 3A, 7A, 8, & 9 (SARS-2 ORF 3A, ORF 7A, ORF8, & ORF9), and papain-like protease (SARS-2 Plpro. **(C)** Analysis of the heatmap data from panel **(A)** revealing the sum of IgM NFI responses for combination of different SARS-CoV-2 spike antigens such as SARS-CoV-2 S1, S2, and RBD subunits. In panels **(B, C)**, PBS and TSP treated samples were represented in blue and red dots, respectively. Data was expressed as median ± 95% CI and the statistical inferences was derived using nested one-way ANOVA with Sidak’s multiple comparison *post-hoc* analysis (**** p < 0.0001, ns – not significant, as indicated). Co-mV, COVID-19 mRNA vaccine; PBS, phosphate buffered saline; TSP, tacrolimus-sirolimus-prednisone combination; IgM, immunoglobulin M; CI; confidence interval; NFI, normalized fluorescence intensity.

Pre-immunization or day 1 following the prime dose of Co-mV did not produce any IgM antibody response against different SARS-CoV-2 proteins, as expected (**Figure 5A**; PBS). Analysis of the heatmap data indicated that there was no significant change in IgM antibody levels against a combination of different domains devoid of spike domains (**Figure 5B**; PBS). However, a remarkable elicitation of IgM antibody levels against combined SARS-CoV-2 S1, S2, and RBD subunits was noted in the 5-day post-immunization sera (**Figure 5C**; PBS). The increased levels in the combined spike IgM levels stayed stable for 10 days, which, as expected, waned at the end of 21 days following prime dose vaccination (**Figure 5C**; PBS). Interestingly, the IgM response on day 26, which is day 5 past 2D immunization (on 21^st^ day) was found to be nearly doubled compared to day 5 after prime immunization (**Figure 5C**; day 5 vs 26; PBS). These trends of waning of responses after first dose and pronounced response following 2D reflected the typical human immune responses observed for Co-mV (23). Strikingly, the TSP pretreatment completely attenuated the IgM levels against a combination of different spike domains at all the time points tested (**Figure 5C**). In other words, the responses elicited in the TSP-pretreated group were found to be similar to that of the pre-immunization state (**Figures 5A, C**, day 26 vs day -4).

**Figure 6:**
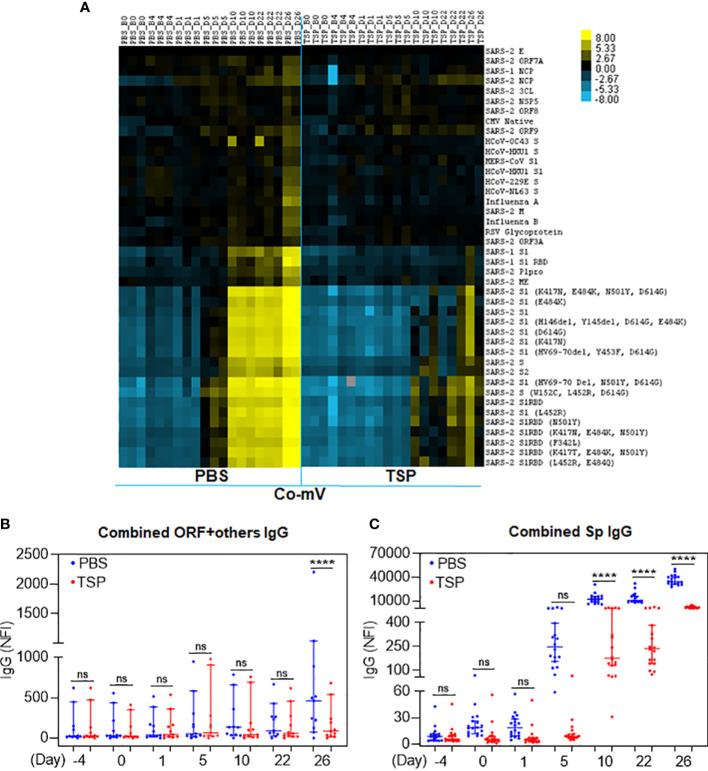
TSP drug combination attenuated the Co-mV- production of IgG antibodies against SARS-CoV-2 viral antigens in Balb/C mice. IgG antibody profile to SARS-CoV-2 viral antigens in the PBS vehicle treated and TSP drug combination treated Balb/C mice that were immunized with Pfizer Co-mV, as indicated in materials and methods. **(A)** The heatmap shows IgG reactivity expressed in terms of row z-score for a respective antigen across different mice samples. Each antigen was organized into rows with serum specimens organized into columns classified as B0, B4, D1, D5, D10, D22, and D26 (similar to that detailed in **Figure 5**). Reactivity was represented by color (Light Blue = low, Black = mid, Yellow = high) and the heatmap represents NFI as detailed in **Figure 5**. **(B)** Analysis of heatmap data illustrating the sum of IgG NFI responses for combination of SARS-CoV-2 antigens representing different domains devoid of spike as detailed in panel **(B)** of **Figure 5**. **(C)** Analysis of the heatmap data revealing the sum of IgG NFI responses for combination of different SARS-CoV-2 spike antigens such as SARS-CoV-2 S1, S2, and RBD subunits. In panels **(B, C)**, PBS and TSP treated samples were represented in blue and red dots, respectively. Data was expressed as median ± 95% CI and the statistical inferences was derived using nested one-way ANOVA with Sidak’s multiple comparison *post-hoc* analysis (**** p < 0.0001, ns – not significant, as indicated). Co-mV, COVID-19 mRNA vaccine; PBS, phosphate buffered saline; TSP, tacrolimus-sirolimus-prednisone combination; IgG, immunoglobulin G; NFI, normalized fluorescence intensity; CI, confidence interval.

For IgG responses, the heat map shows distinct clustering of seropositive responses to the SARS-CoV-2 subunits in the vaccine alone group irrespective of the number of doses (**Figure 6A**, PBS). In particular, the IgG reactivities against most of the spike domains were found to be prominent at day 10 following the prime dose vaccination, that remained stable throughout the period past the priming dose and until the time of 2D vaccination (**Figures 6A, C**). It is to be noted that the prominent period of IgG emergence preceded by the IgM reactivities (at day 5 after dose 1) and the elicited IgG response did not appear to wane unlike the IgM response pattern. This *in vivo* pattern clearly reflected the classical immunological phenomenon that IgM antibodies are the first antibodies produced during an immune response, which also declined more rapidly than IgG antibodies. In contrast, the TSP pretreatment exhibited a robust suppression of the Co-mV-induced serum IgG reactivities to the combined SARS-CoV-2 subunits (**Figure 6C**; p<0.0001). Even the later time point, day 26 corresponding to 5-day post booster immunization resulted in a muted response (**Figure 6C**; TSP vs PBS). There was no noticeable change in IgG antibody levels against a combination of different domains devoid of spike domains except day 26 (**Figure 6B**; PBS). This *in vivo* data is pertinent concerning the clinical settings that a commonly used ISs combination for the organ transplantation can severely dampen the Co-mV-induced antibody response, particularly even after repeated boosting.

### Effect of TSP combo treatment on the levels of SARS-CoV-2 Sp immunogen *in vivo*

**Figure 7:**
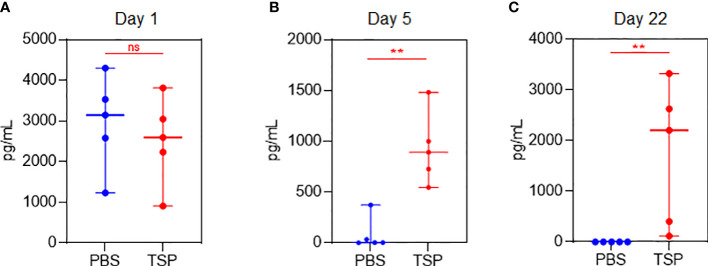
TSP drug combination over time differentially regulated Co-mV-induced production of SARS-CoV-2 spike antigen levels in Balb/C mice. Sandwich ELISA (as described in materials and methods) to assess the SARS-CoV-2 spike protein antigen in the non-hemolyzed sera of vaccine alone and TSP-pretreated vaccine group using 5 samples from each group on **(A)** day 1, **(B)** day 5, and **(C)** day 22. In panels **(A-C)**, the data was represented as median values ± 95% CI; blue indicates PBS group and red indicates TSP group. Statistical comparisons were performed using Mann-Whitney test (** p = 0.004; ns, not significant). Co-mV, COVID-19 mRNA vaccine; PBS, phosphate buffered saline; T, tacrolimus; S, sirolimus; P, prednisone; CI, confidence interval.

Both Pfizer and Moderna Co-mV use nucleoside-modified mRNA with a lipid nanoparticle-formulation to encode the Sp protein of SARS-CoV-2. Thus, to understand whether the translational capacity of the mRNA from Co-mV to produce S protein *in vivo* is hampered by a commonly used ISs combo used in organ transplantation, we used a sandwich ELISA to assess the SARS-CoV-2 Sp immunogen in the Balb/c mice sera of vaccine alone and TSP-pretreated vaccine groups. As expected, following vaccination, the day 1 sera had a higher level of Sp Ag in the absence of any IS treatment (compare PBS of day 1 with PBS of days 5 and 22). The TSP-pretreatment resulted in a decreasing trend of the mean Sp immunogen levels when compared to the vaccine alone group at day 1 post first dose vaccination (**Figure 7A**). This points out that TSP can at least partially impede the translation of Sp immunogen. Since mRNA translation begins immediately after vaccine inoculation, serial sampling within day 1 would have been more informative, which is not studied (due to practical reasons to limit frequent draws) and is one of the limitations of this study. Further, the Sp immunogen levels on day 5 after first dose vaccination were found to be surprisingly elevated that was further increased on day 22, which is 1-day after the second dose in TSP group (**Figure 7B, C**). Although this inverse trend in the antigen and antibody level at or beyond day 5 appears to be intriguing, it might have resulted from a poor clearance of the already produced Sp antigen owing to the inability of neutralizing antibody production (43, 44). Also, this sandwich assay typically captures and quantifies the free Sp immunogen, and given the cross-section of analysis, the negligible antibody levels observed due to TSP pretreatment might have allowed more free Sp immunogens to be captured and detected by the assay. Nevertheless, the *in vivo* antigen data hints at the TSP-induced diminution of Co-mV response could be resultant of repressed translation, at least partially.

### Temporary drug holiday of sirolimus and not using higher concentrations of Co-mV rescued the ISs-induced translational repression of Sp immunogen

**Figure 8:**
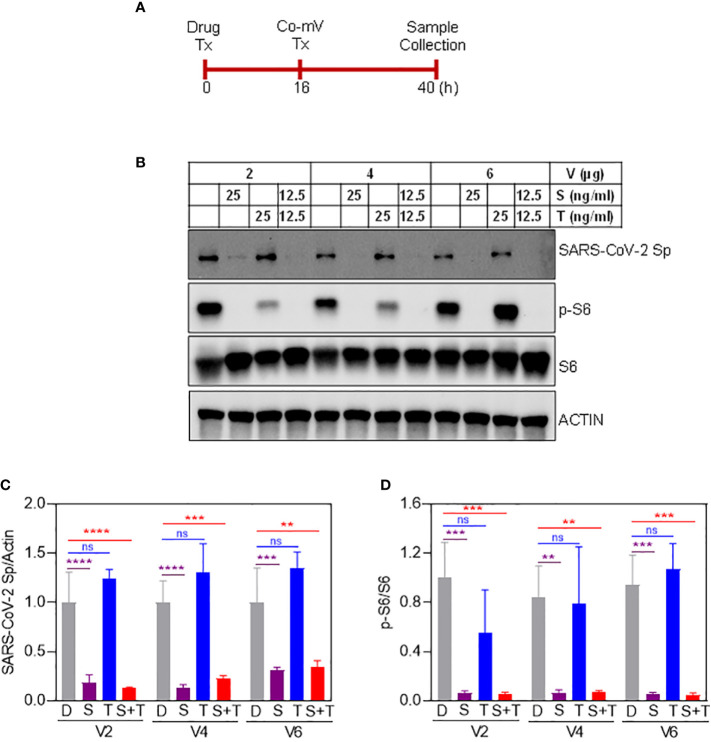
Increased dose of vaccine did not overcome the effect of sirolimus-induced repression of spike protein expression in Balb/C mice. **(A)** Schematic illustration of drug and Co-mV transfection schedule to test whether an increasing dose of vaccine rescues sirolimus-induced repression of Sp protein expression. **(B)** As indicated, HEK293 cells were pretreated with the drugs, S and T either individually or in combination at the indicated concentrations for 16 h followed by which increasing concentrations (2, 4, and 6 μg) of Pfizer-BioNTech Co-mV was transfected in the presence of drugs for an additional 24h. After the experimental period, protein was extracted from the cells and an equal amount of protein from all the groups were analyzed for the expression of SARS-CoV-2 Sp protein, phospho-specific protein S6, total S6, and the loading control, beta-actin (as indicated in materials and methods). **(C, D)** Quantification of the immunoblots in panel **(A)** expressed as a ratio of SARS-CoV-2 Sp to Actin; and phospho-specific protein S6 to total S6, respectively. Relative values to DMSO controls were shown. Data was expressed as the mean ± SD (n=3) and the statistical inferences between DMSO and treatment groups were made from a two-way ANOVA, Tukey *post-hoc* multiple comparisons. (In panel **(C)**, ** p = 0.001; *** p = 0.0002 (V4 – D vs S+T); *** p = 0.0006 (V6 – D vs S); **** p < 0.0001; In panel **(D)**, ** p = 0.0011 (V4 – D vs S); ** p = 0.0012 (V4 – D vs S+T); *** p = 0.0001 (V2 – D vs S and D vs S+T); *** p = 0.0003 (V6 – D vs S); *** p = 0.0002 (V6 – D vs S+T), as indicated). Co-mV, COVID-19 mRNA vaccine; Tx, treatment; V0, no CO-mV; V2, 2 μg Co-mV; V4, 4 μg Co-mV; V6, 6 μg Co-mV; D, dimethyl sulfoxide (DMSO); S, sirolimus; T, tacrolimus; SARS-CoV-2 Sp, severe acute respiratory syndrome coronavirus 2 spike; S6, S6 ribosomal protein; pS6, phospho-S6 ribosomal protein; β-actin, beta-actin.

**Figure 9:**
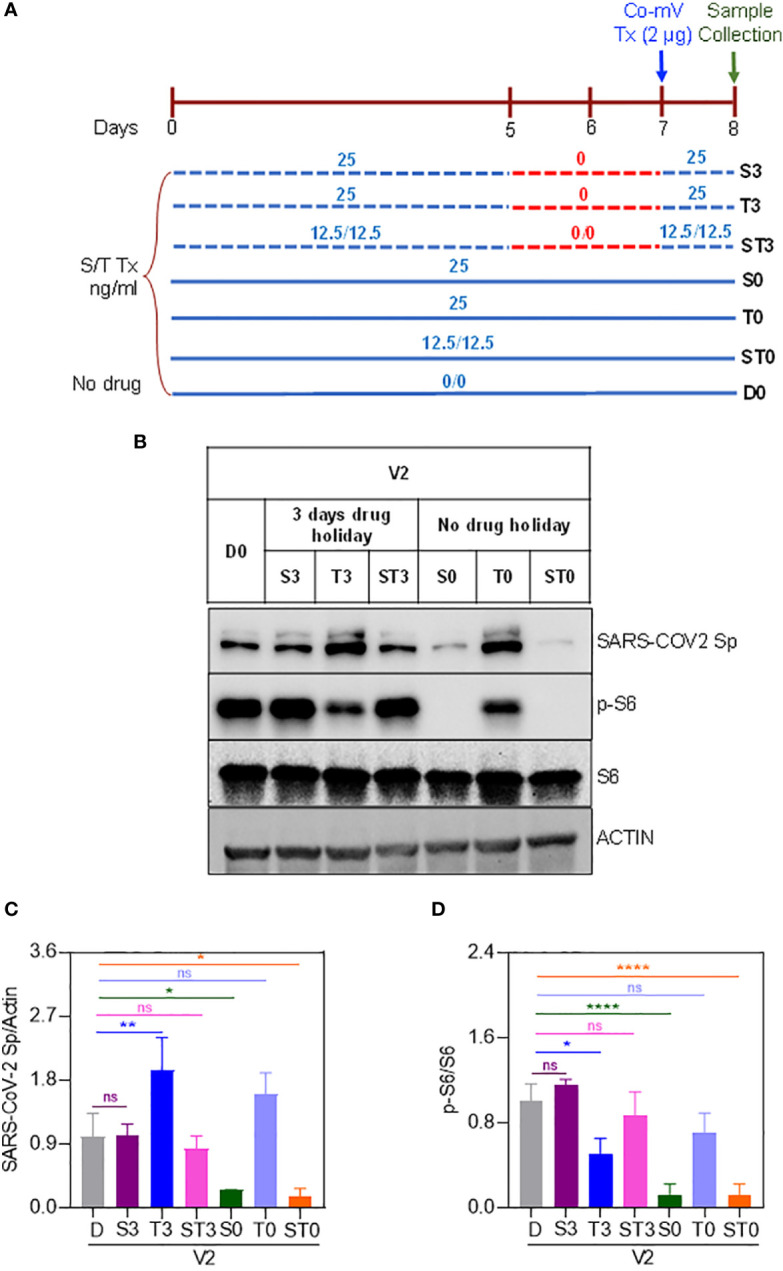
Three-days complete holiday of drug(s) rescued the drug-induced repression of spike protein expression in Co-mV transfected HEK293 cells *in vitro*. **(A)** 8-day experimental schema to assess three-day complete holiday of drugs, which included the following 7 groups: D0, S0, T0, ST0, S3, T3, and ST3. **(B)** The drug combinations were pretreated and maintained for 8 days except for 3-days complete holiday starting day 5 through day 7. All the groups received 2 μg of Pfizer BioNTech Co-mV on the 7^th^ day. The individual concentrations of T and S and their combinations were 25, 25, and 12.5 ng/mL each, respectively. At the end of 8^th^ day, protein lysates were collected from all the groups, and Western analysis were performed for the expression of SARS-CoV-2 Sp, pS6, S6, and β-actin antibodies. A representative immunoblot from three independent experiments was shown. (C & D) Quantification of the immunoblots in panel **(A)** expressed as a ratio of SARS-CoV-2 Sp to Actin; and phospho-specific protein S6 to total S6, respectively. Relative values to DMSO controls were shown. Data was expressed as the mean ± SD (n=3) and the statistical inferences between DMSO and treatment groups were made from one-way ANOVA, Tukey *post-hoc* multiple comparisons. (In panel **(C)**, * p = 0.04 (D vs S0); * p = 0.02 (D vs ST0); ** p = 0.009; In panel **(D)**, * p = 0.02 (D vs T3); **** p < 0.001, ns – not significant, as indicated). Co-mV, COVID-19 mRNA vaccine; V2, 2 μg of Pfizer BioNTech Co-mV; S, sirolimus; T, tacrolimus; Tx, treatment; Group D0, DMSO and no drug; Group S0, Tx with only S for 8 days with no interruption; Group T0, Tx with only T without drug stoppage; ST0, Tx with S and T combination without drug holidays; Group S3, Tx with only S with 3 days drug stoppage; Group T3, Drug T with a transient halt of drug treatment for 3 days; ST3, Tx with S and T combination with a continuous interruption of drugs for 3 days; SARS-CoV-2 Sp, severe acute respiratory syndrome coronavirus 2 spike; S6, S6 ribosomal protein; pS6, phospho-S6 ribosomal protein.

While a significant proportion of SOT recipients reported to remain as poor responders even after receiving 3 or more doses of Co-mV, optimization strategies are required to improve the vaccine response in at-risk poor responders in IC groups. To this end, we next explored any means of rescuing and/or improving the translational capacity of Co-mV in the presence of ISs *in vitro*. To test this *in vitro*, we first assessed whether higher concentrations of Co-mV could overcome the ISs, specifically sirolimus-induced translational suppression of Sp immunogen. Increased doses up to 6 μg of Co-mV did not rescue the sirolimus-inhibited Sp expression and p-S6 activation (**Figures 8A–D**). Further, 1-day reduced concentration of sirolimus was also not effective in rescuing the ISs-induced repression of Sp immunogen and translational capacity determined in terms of p-S6 activation (**Figure S6**). Next, the impact of transient discontinuation of S in TSP combination on Co-mV translation was assessed. 3-days but not 1-day temporary drug holiday of S in the TSP combination exhibited a restoration of translational repression of Sp immunogen with a concomitant restoration in the translational capacity as measured by p-S6 levels (**Figures 9A–D**). These data indicate that neither higher concentrations of Co-mV nor 1-day reduced concentration of ISs could overcome the sirolimus-induced inhibition of Sp protein expression and translational capacity. But a complete drug holiday of sirolimus for 3-days or selective switch to tacrolimus has the potential to rescue Sp levels concomitant with the translational capacity *in vitro*. However, detailed pre-clinical and clinical validations are warranted to translate these *in vitro* strategies.

## Discussion

We and others have reported that Co-mV generates poor immunological response in IC patients as in SOT, AI disorder, and CIDs (1–6). Majority of these studies have illustrated that the poor Co-mV-driven responses are at least partly linked to the use of immunosuppressive class of medicines that inhibit the humoral and cell-mediated immune processes. Notably, such investigations have precluded the ‘empirical understanding’ of mRNA translational efficiency, a rate-limiting mechanism that can dictate the efficacy of new-generation mRNA vaccine. This study uses *in vitro*, *in vivo*, and supporting clinical data and interrogates the effect of ISs on the translation of Co-mV, which is central to its effectiveness.

In our study, irrespective of 2D or 3D Co-mV immunizations, IC patients exhibited poor antibody response compared to healthy or non-transplanted participants. In addition, the neutralizing IgG titer threshold of 4160 AU/mL according to Ebinger et al. (40) was found to be consistently low in IC patients compared to the healthy controls (**Table 2**). Adjusting for the differences across IC patients receiving ISs therapies particularly with mycophenolate or sirolimus, our finding indicates that these ISs used in the treatment of transplant and autoimmune patients, can inhibit the 2D Co-mV-evoked initial production of IgM (the first antibody to develop as part of humoral immune response) that subsequently reflected on the IgG levels as well.

A diminished antigen level (because of translation) could be a plausible reason in the non-responders. In a small-sampled analysis, we have earlier shown a direct relationship between Sp Ag level and humoral immunogenicity in healthy volunteers following the first dose of Moderna mRNA vaccination (44). Relevantly, when we link and extrapolate these findings to the IC dataset, it can be reasonably presumed that a low Ag level could be a cause for poor antibody response to Co-mV in IC patients that are on ISs. Our antigen/antibody binning data according to Sp Ag thresholds, points out that impairment in Sp immunogen production (associated with translation process) *per se* may only have a partial link to the Co-mV-induced muted antibody response since we observed only a decreasing trend in Sp immunogen, albeit non-significant in IC patients relative to HC. This can be attributed to the fact that Sp Ag levels were not measured in all samples. Since, translation capacity is tightly coupled to nutrient availability, metabolism status, severity of the disease/stress, choice and chronicity of drug treatments, aging, and genetics, etc., a more controlled study accounting for these factors can help better understand the relevance of translation. Another reason for this contrast observation could be due to the fact that a majority of the samples tested were collected 10 or more days after the first dose, when the Ag levels diminished from the peak level (44). Indeed, to show that restrained Sp antigen levels as a resultant of translation inhibition is underlying the poor Co-mV response in IC setting, it is desirable to quantify Sp antibody along with Sp antigen in the early stages post-first vaccination as mRNA translation begins immediately after vaccine inoculation. Because the later periods and subsequent exposures might trigger other memory/immune-associated mechanisms and would render it complex to guage the precise role of translation. Since the large majority of high-risk IC cohorts are already being boosted with 3 or more doses, this left a bottleneck in obtaining early samples following the first dose of Co-mV (around the peak Ag and Ab period). Thus, our clinical data should be regarded as a support to hypothesis generation and only a basis for testing our hypothesis on the role of translation *in vitro* and *in vivo*.

So far, the poor Co-mV immunogenicity associated with several conditions, including IC has been mostly associated with immune modulation by ISs and only theorized to result from differences in translational efficiency without empirical demonstration. In the context of experimental evaluation, our *in vitro* findings suggest that the most widely used ISs in SOT patients, namely, mycophenolate followed by sirolimus, can significantly attenuate the Co-mV-induced Sp immunogen levels. This paralleled with the ISs-induced repression of translational capacity (measured in terms of p-S6 phosphorylation) but in the order of sirolimus followed by mycophenolate. This suggests that M can influence a process related to translation of Sp antigen that are not involving classical cellular signaling. For instance, M has been shown to trigger nucleolar stress *via* disrupting the production and structural integrity of ribosomes, which are macromolecular machines responsible for protein synthesis in the cell (21). Consistent with our findings, the ISs such as T, M, S, P and/or its combination (TMP/TSP) have been reported to impact the translation process (protein synthesis) either directly or indirectly (12, 15–17, 19–22), which is sufficient to disrupt the production of Sp protein. Interestingly, our *in vitro* data also indicates that the TSP-induced low protein expression of Sp immunogen did not result from deficient Co-mV uptake and its associated Sp mRNA expression. Genetic and pharmacological manipulation-based gain-of-function experiments involving components of translation process (such as mTOR, pS6, 4EBP1 etc.), however, will provide an unambiguous role for translation process impacted by ISs.

Validating our *in vitro* data, the immunization of Balb/c mice with Co-mV when pretreated with TSP clearly exhibited negligible to modest IgM and IgG seropositive responses to the SARS-CoV-2 subunits compared to the vehicle alone treated vaccine group. The modest seropositive response was seen only in the case of IgG response after the second dose of Co-mV. Concomitantly, we observed that the TSP-pretreatment resulted in a decreasing trend of the mean Sp immunogen levels, however not significant, when compared to the vaccine alone group as early as day 1 following the first dose vaccination (**Figure 7A**). Surprisingly, we found the Sp immunogen levels on day 5 after first dose vaccination to be elevated in the TSP group that was further increased on day 22 (a day after the second dose) (**Figure 7B, C**). This data likely indicates that there is a lack of adequate processing of already produced Sp Ag into the subsequent steps *viz.* Ag presentation, which is reflected by bare minimal antibody levels (both IgM and IgG; **Figure 5** and **6**). Alternatively, given the concentration of an Ag relies on a balance between Ag production and its clearance by endocytosis and lysosomal degradation, a relatively low production of TSP-induced Ag noted on day 1 (Veh vs TSP; **Figure 7A**) might have sedated its removal. In line with this claim, we have earlier shown clear evidence for a negative relationship between antigen clearance and anti-SARS-CoV-2 antibody production in COVID-19 patient samples (43). Overall, our *in vivo* data alludes TSP may at least partially repress the translation of Sp immunogen affecting the immunogenic response to COVID-19 vaccination. Prior studies have shown that sirolimus can abrogate the immune responses by inhibiting mTOR-dependent protein synthesis in immune cells (45). Also, prednisone using rheumatoid arthritis IC patients showed a markedly depressed rate of muscle protein synthesis (46). Since the IC patients use several classes of ISs, in-depth studies are warranted to understand the influence of other commonly used drugs or its combinations on the translational process and its associated antibody responses from mRNA-based vaccines.

Exploring any means of rescuing and/or improving the translational capacity of Co-mV in the presence of ISs, we observed that neither higher concentrations of Co-mV up to 6 μg nor reduced concentration of S for a single day could overcome the translational suppression of Sp immunogen and S-induced muted translation *in vitro*. Interestingly, 3-day temporary drug holiday of S in the TSP combination exhibited a restoration of protein levels of Sp immunogen with a concomitant reinstatement of the translational capacity. Although this temporary *in vitro* complete drug holiday presents early excitement, we must consider the translatability of temporary suspension of ISs like sirolimus in the IC patients. Likewise, our findings advocate selective switching of ISs to tacrolimus that can maintain the immunosuppressive activity while exerting a less impact on the mRNA translational process and to augment vaccine response.

In this study, we report ISs used by the IC patients can dampen the translation process and contribute to poor Co-mV efficacy. Manipulating the appropriate combination of ISs during Co-mV period may contribute to long lasting vaccine efficacy in IC patients. Thus, in the grand scheme, it seems prudent to err on the side of caution by considering the ‘translation regulation (here, activation)’ when prioritizing new generation mRNA-based treatments to the high-risk IC groups that are on ISs. Future studies such as this should galvanize the scientific community to delve deep into the understanding and tailoring the choice of ISs therapy with respect to translation for a better outcome of COVID-19 mRNA vaccines.

## Data availability statement

The original contributions presented in the study are included in the article/**Supplementary Material**. Further inquiries can be directed to the corresponding authors.

## Ethics statement

The studies involving human participants were reviewed and approved by Institutional review board of the University of Texas Southwestern Medical Center approved this study. Written informed consent for participation was not required for this study in accordance with the national legislation and the institutional requirements. The animal study was reviewed and approved by The UT Southwestern Institutional Animal Care and Use Committee (IACUC).

## Author contributions

NM, LM, and AM conceived the idea, provided overall direction, planning, and wrote the original draft of the manuscript. KK, NM, and Q-ZL performed the experiments and contributed to the acquisition of the data. BG and DG provided two-dose healthy control and immunocompromised clinical samples and related clinical information. EA, RZ, and LM extracted and arranged electronic health data from EPIC for analysis. EK and WT provided vaccines and test drugs. C-AC shared their data/sample set for internal comparisons/validations, participated in discussions, and provided critical inputs. SM provided research personnel, laboratory support, and other resources to accomplish all the basic science experiments. NM, LM, KK, SM, and AM helped troubleshoot the experiments and verified the results. SM and AM contributed to the implementation and supervision of the work. NM, LM, BG, DG, SM, and AM contributed to the review, editing, and drafting of the final version of the manuscript. All authors contributed to the article and approved the submitted version.

## Funding

This work was supported by grants from CPRIT (RR170003) to SM.

## Acknowledgments

The authors thank David R Walt, Harvard Medical School and Brigham and Women’s Hospital, Department of Pathology, Boston, MA for his insightful inputs. We also thank Donglu Xie, Programmer/Architect Lead, Information Resources-Academic and Administration Informatics resources (IR-AAIR), UT Southwestern Medical Center for 2D vaccine data pull. We thank Mauricio Marquez-Palencia and Pravat Kumar Parida for their assistance with animal experiments. We thank Abbott Diagnostics Division (IL, USA) for providing us SARS-CoV-2 specific antibody test kits to validate the assays and further clinical evaluations. Abbott Diagnostics, while provided part of the reagents for antibody testing, did not have any role in the study’s design, collection, analyses, or interpretation of data; drafting manuscript, or in the decision to publish the results.

## Conflict of interest

The authors declare that the research was conducted in the absence of any commercial or financial relationships that could be constructed as a potential conflict of interest.

## Publisher’s note

All claims expressed in this article are solely those of the authors and do not necessarily represent those of their affiliated organizations, or those of the publisher, the editors and the reviewers. Any product that may be evaluated in this article, or claim that may be made by its manufacturer, is not guaranteed or endorsed by the publisher.

## Supplementary material

Supplementary Figure 1 Flow chart of cases used in the COVID-19 mRNA vaccine experiments. (A) A total of fifty healthy controls (HC) and 259 immunocompromised (IC) patients on immunosuppressive (ISs) drugs were included in the assessment of humoral (IgM_Sp_ and IgG_Sp_) responses to 2 doses of mRNA vaccines. Out of 50 total HC and 259 IC patient samples, 16 and 174, respectively, were only assessed for both the spike antigen (Ag) and antibody (Ab) levels. (B) Flow chart to assess the influence of 3 doses of COVID-19 vaccine on spike IgG_Sp_ antibody levels in 843 non-transplanted controls (NT) and 67 solid organ transplanted IC patients on ISs.

Supplementary Figure 2 Immunocompromised SOT patients after 3 doses of COVID-19 vaccines showed poor IgG_Sp_ response compared to non-transplanted subjects. Figure shows IgG_Sp_ levels in NT subjects and immunocompromised SOT patients on ISs who received 3D COVID-19 vaccines. Box plot indicates the mean ± SD of IgG_Sp_ (AU/mL) levels. Comparison of the means among the groups and the statistical difference was established using a two-tailed Mann-Whitney test. NT, non-transplanted; SOT, solid organ transplanted; ISs, immunosuppressants; 3D, three doses; Sp, spike; AU, arbitrary units; SD, standard deviation; IgG, immunoglobulin G; ↓, decrease.

Supplementary Figure 3 Optimization of vaccine transfection and spike protein expression *in vitro* in HEK293 cells. (A) Different concentrations (1, 2, and 3 μg) of Pfizer-BioNTech Co-mV was transfected into HEK293 cells with and without lipofectamine. At the end of 24h, protein was extracted from the cells and an equal amount of protein from non-transfected and transfected lysates were analyzed using immunoblotting (as indicated in materials and methods) for the expression of SARS-CoV-2 Sp protein using a specific primary antibody for SARS-CoV-2 spike. The same immunoblots were stripped and reprobed with an actin antibody which was used as loading control. (B) Choosing an optimal concentration of 2 μg Pfizer Co-mV and without lipofectamine, time-course analysis was performed on HEK293 cells with transfection carried out for different time points, as indicated. Western analysis for SARS-CoV-2 Sp protein was performed and representative full immunoblot was shown. (C) The full immunoblot membrane processed as in panel B was subsequently stripped and reprobed for actin, as loading control. SARS-CoV-2 Sp, severe acute respiratory syndrome coronavirus 2 spike; Co-mV, COVID-19 mRNA vaccine; HEK293, human embryonic kidney 293.

Supplementary Figure 4 Confirmation of uptake of vaccine spike mRNA by HEK293 cells treated with Pfizer-BioNTech Co-mV *in vitro*. (A-D) HEK293 cells were transfected with 2 μg Pfizer Co-mV without lipofectamine. RNA was purified after thoroughly washing the cells with sterile PBS and real-time qRT-PCR was performed using four different primer sets targeting four different locations in Pfizer-BioNTech Co-mV (as provided in materials and methods). RNA levels of spike were presented as 2^−ΔΔCT^ values relative to house-keeping gene, actin. Results are from four independent experiments (n = 4). Differences between the non-transfected and transfected groups were analyzed using a two-tailed Welch’s t-test. Data was expressed as the mean ± SD. (*** p = 0.001 (Figure S4A); **** p < 0.0001 (Figure S4B); *** p = 0.0009 (Figure S4C); **** p < 0.0001 (Figure S4D) *vs* non-transfected controls, as indicated). Co-mV, COVID-19 mRNA vaccine; PBS, phosphate buffered saline; qRT-PCR, real-time quantitative reverse transcription-polymerase chain reaction; SD, standard deviation; CT, cycle threshold; 2^−ΔΔCT^, 2ˆ(–delta delta CT), a comparative CT method quantification.

Supplementary Figure 5 Immunosuppressant combination TSP-associated body weight changes in Balb/C mice following immunization with Pfizer Co-mV. 8-10 weeks old healthy BALB/c female mice (n = 5) were immunized twice three weeks apart with 2 µg of Pfizer-BioNTech Co-mV formulation intramuscularly. Drug combination of TSP treatment was started 7 days prior to Co-mV administration and continued through the entire experimental period daily by oral gavage according to their body weight. The individual concentrations of T, S, and P drugs were 12.5, 12.5, and 5 mg/kg, respectively. PBS served as vehicle control for the drug injections (n=5). Body weight was measured every 7 days and data was expressed as mean ± SD. Statistical comparisons were performed using Kruskal-Wallis One-Way ANOVA. Co-mV, COVID-19 mRNA vaccine; PBS, phosphate buffered saline; T, tacrolimus; S, sirolimus; P, prednisone; SD, standard deviation; ANOVA, analysis of variance.

Supplementary Figure 6 Reduction of sirolimus and tacrolimus dosage for one day just prior to Co-mV transfection did not rescue the drug-induced repression of spike protein expression *in vitro*. (A) 7-day experimental schema for the drug treatment and vaccine transfection in HEK293 cells to assess reduced dosage of drugs for 1 day prior to Co-mV transfection. This included the following 4 groups: D0, R0, R1, and R2. (B) S and T drug combination each at concentrations of 12.5 ng/mL were pretreated and maintained for 7 days (R0) except for 1-day reduction (day 5 through day 6) to 1.25 ng/mL each (R1 group) and 6.25 ng/mL each (R2 group). D0 served as no drug controls. All the 4 groups were transfected with 2 μg of Pfizer BioNTech vaccine on the 6^th^ day, which were then allowed for another 24h before terminating the experiment. At the end of the 7^th^ day, protein was extracted from the cells and an equal amount of protein lysates from all the groups were analyzed using immunoblotting (as indicated in materials and methods) for the expression of SARS-CoV-2 Sp protein using specific primary antibody for SARS-CoV-2 spike. The same blots were stripped and reprobed with pS6, S6, and β-actin antibodies. Co-mV, COVID-19 mRNA vaccine; V2, 2 μg of Pfizer BioNTech Co-mV; S, sirolimus; T, tacrolimus; SARS-CoV-2 Sp, severe acute respiratory syndrome coronavirus 2 spike; S6, S6 ribosomal protein; pS6, phospho-S6 ribosomal protein.

Supplementary Figure 7 One-day complete holiday of drug(s) did not rescue the drug-induced repression of spike protein expression in Co-mV transfected HEK293 cells *in vitro*. (A) 7-day experimental schema to assess one-day complete holiday of drugs, which included the following 7 groups: D0, T, TP, TMP, T, TP, and TSP. (B) The drug combinations were pretreated and maintained for 7 days except for one day complete holiday starting day 5 through day 6. All the groups received 2 μg of Pfizer BioNTech Co-mV on the 6^th^ day. The individual concentrations of T, S, M, and P in their respective combinations were 12.5, 10, 10, and 10 ng/mL respectively. At the end of the experimental period on the 7^th^ day, protein lysates were collected from all the groups, and Western analysis were performed for the expression of SARS-CoV-2 Sp protein using specific primary antibody for SARS-CoV-2 spike. The same blots were stripped and reprobed with pS6, S6, and β-actin antibodies. Co-mV, COVID-19 mRNA vaccine; V2, 2 μg of Pfizer BioNTech Co-mV; S, sirolimus; T, tacrolimus; M, mycophenolate; P, prednisone; Group D0, no drug; Group T^1^, 1-day holiday for drugs M and P; Group TP^1^, 1-day holiday for only drug M; TMP, 1-day holiday for all 3 drugs T, M, and P; Group T^2^, 1-day holiday for drugs S and P; Group TP^2^, 1-day holiday for only drug S; TSP, 1-day holiday for all 3 drugs T, M, and P; SARS-CoV-2 Sp, severe acute respiratory syndrome coronavirus 2 spike; S6, S6 ribosomal protein; pS6, phospho-S6 ribosomal protein.
