# Supplementary material for: Translation suppression underlies the restrained COVID-19 mRNA vaccine response in the high-risk immunocompromised group
Source: Front Immunol. 2022 Oct 26;13:1020165. doi: 10.3389/fimmu.2022.1020165 (PMC9644109; doi:10.3389/fimmu.2022.1020165)
Supplement: Supplementary Figure 1 — Flow chart of cases used in the COVID-19 mRNA vaccine experiments. (A) A total of fifty healthy controls (HC) and 259 immunocompromised (IC) patients on immunosuppressive (ISs) drugs were included in the assessment of humoral (IgMSp and IgGSp) responses to 2 doses of mRNA vaccines. Out of 50 total HC and 259 IC patient samples, 16 and 174, respectively, were only assessed for both the spike antigen (Ag) and antibody (Ab) levels. (B) Flow chart to assess the influence of 3 doses of COVID-19 vaccine on spike IgGSp antibody levels in 843 non-transplanted controls (NT) and 67 solid organ transplanted IC patients on ISs. [file Presentation_1.pptx]

## Slide 1
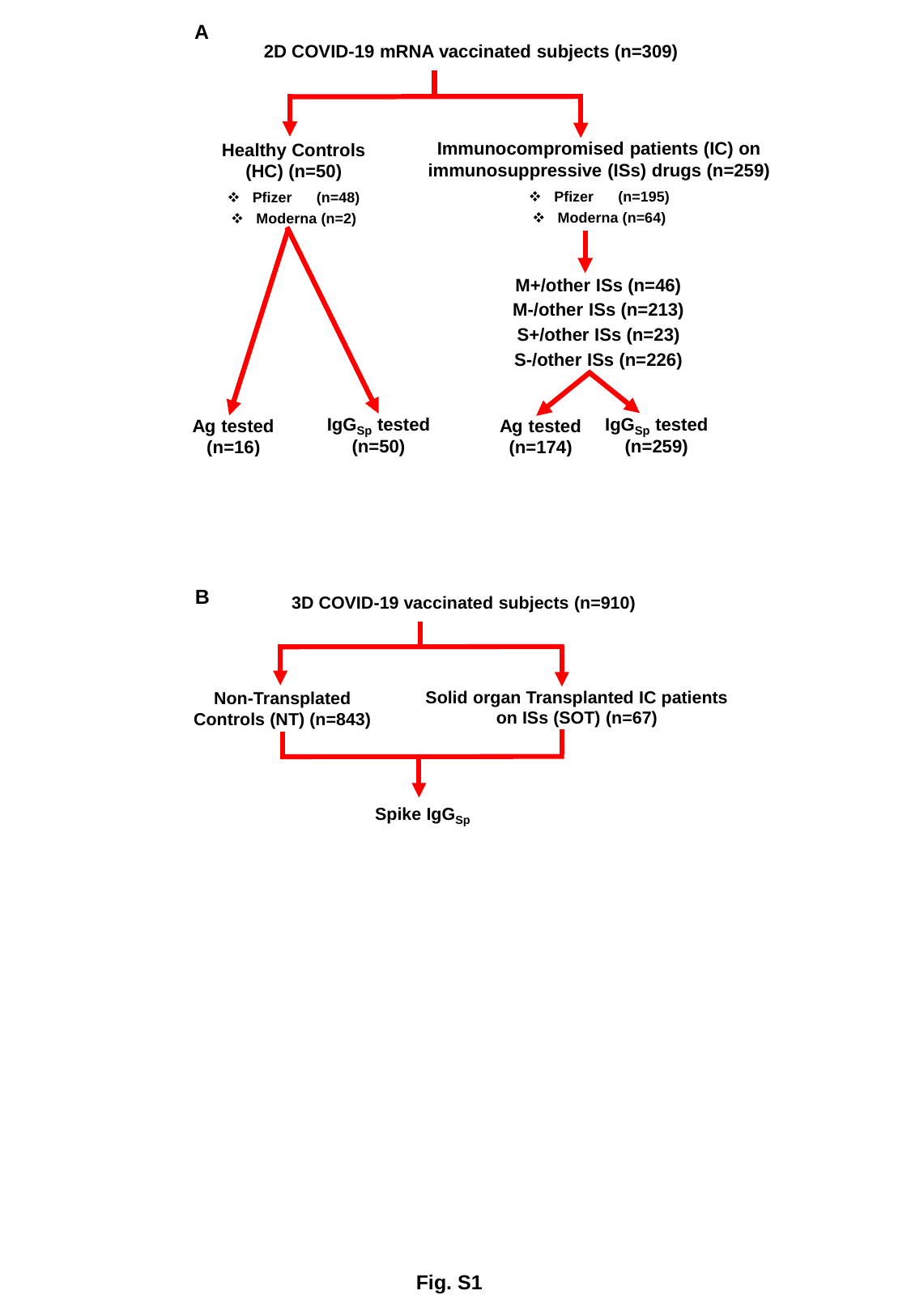

A
B
Fig. S1

## Slide 2
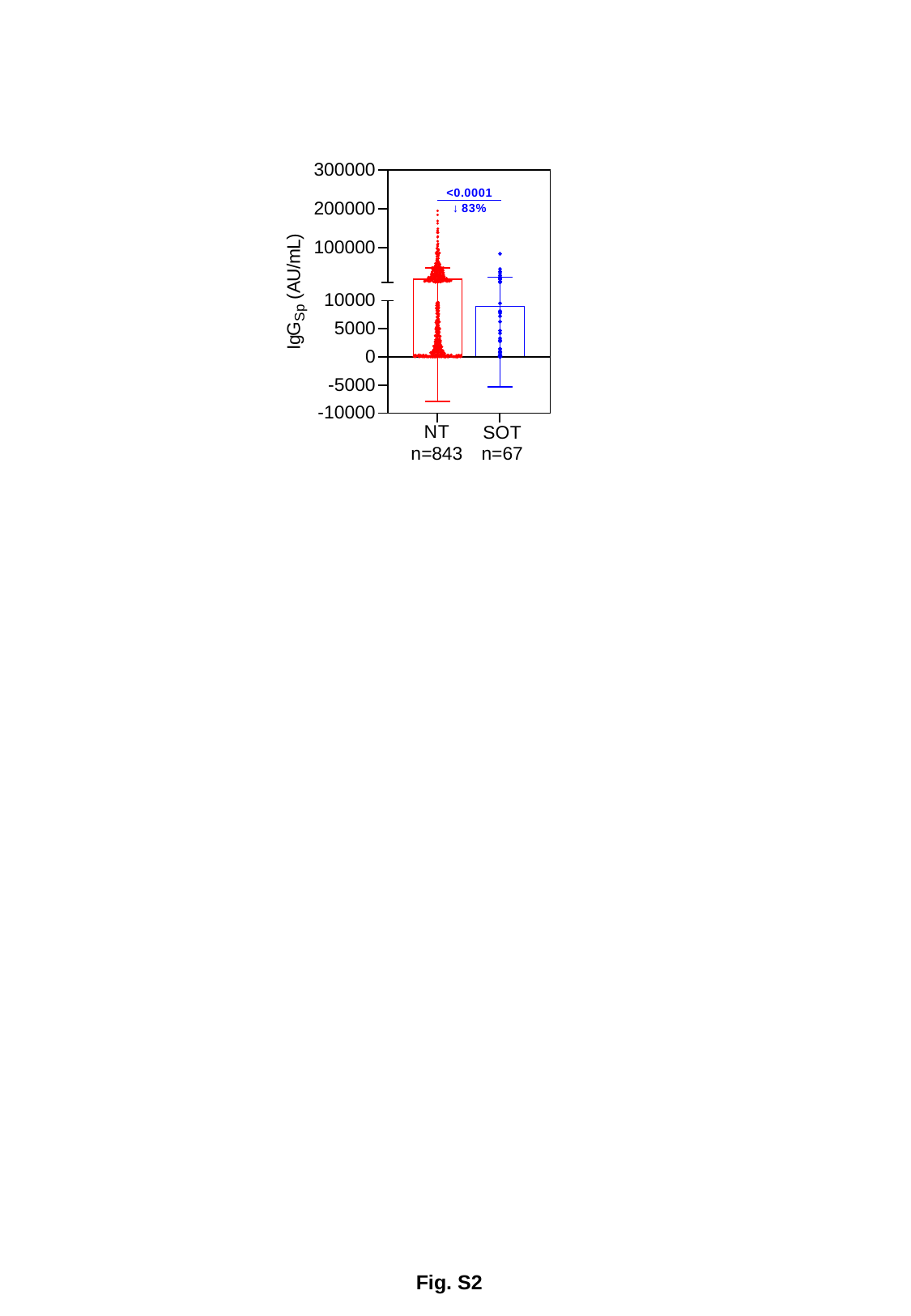

Fig. S2

## Slide 3
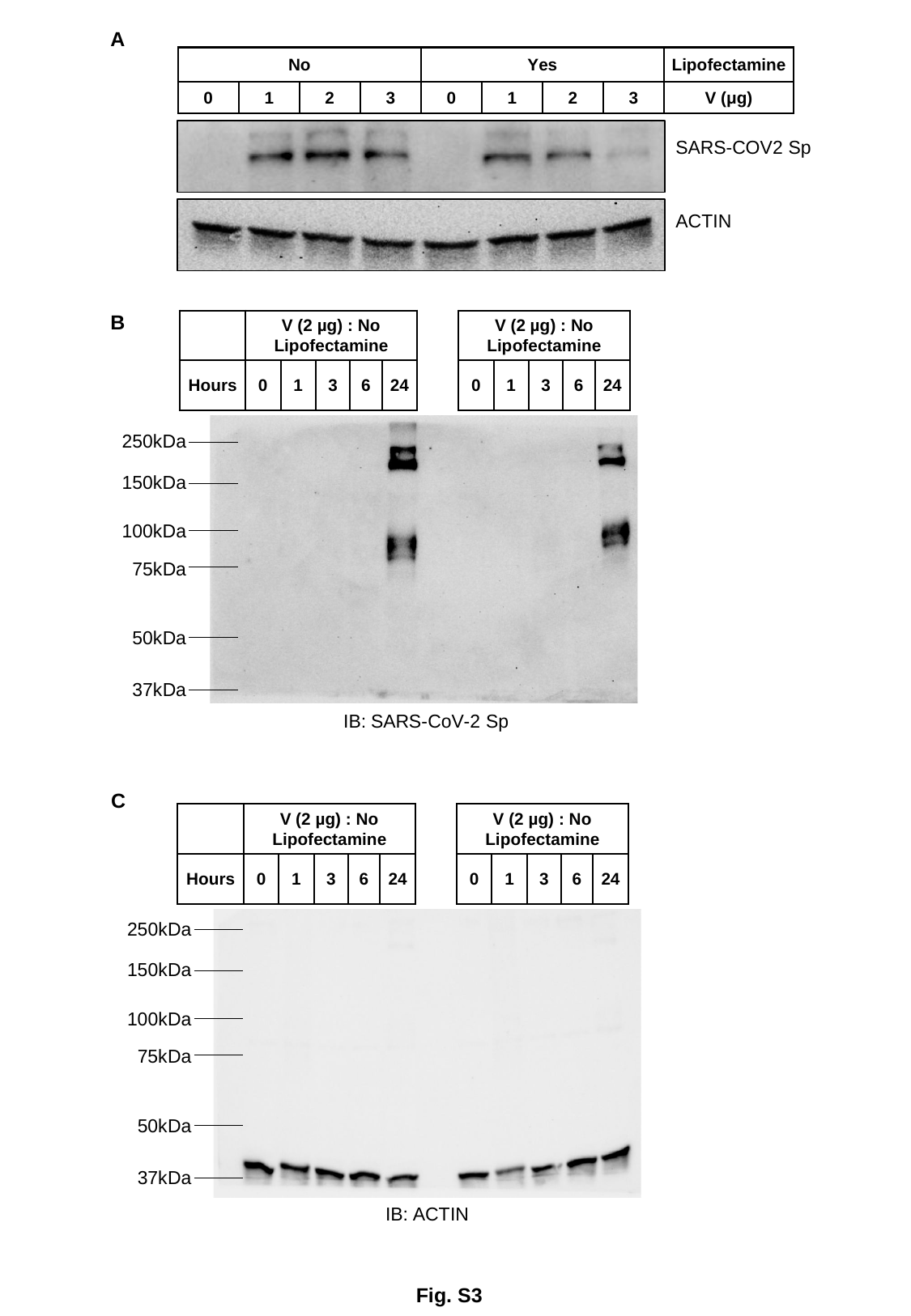

A
B
C
Fig. S3

## Slide 4
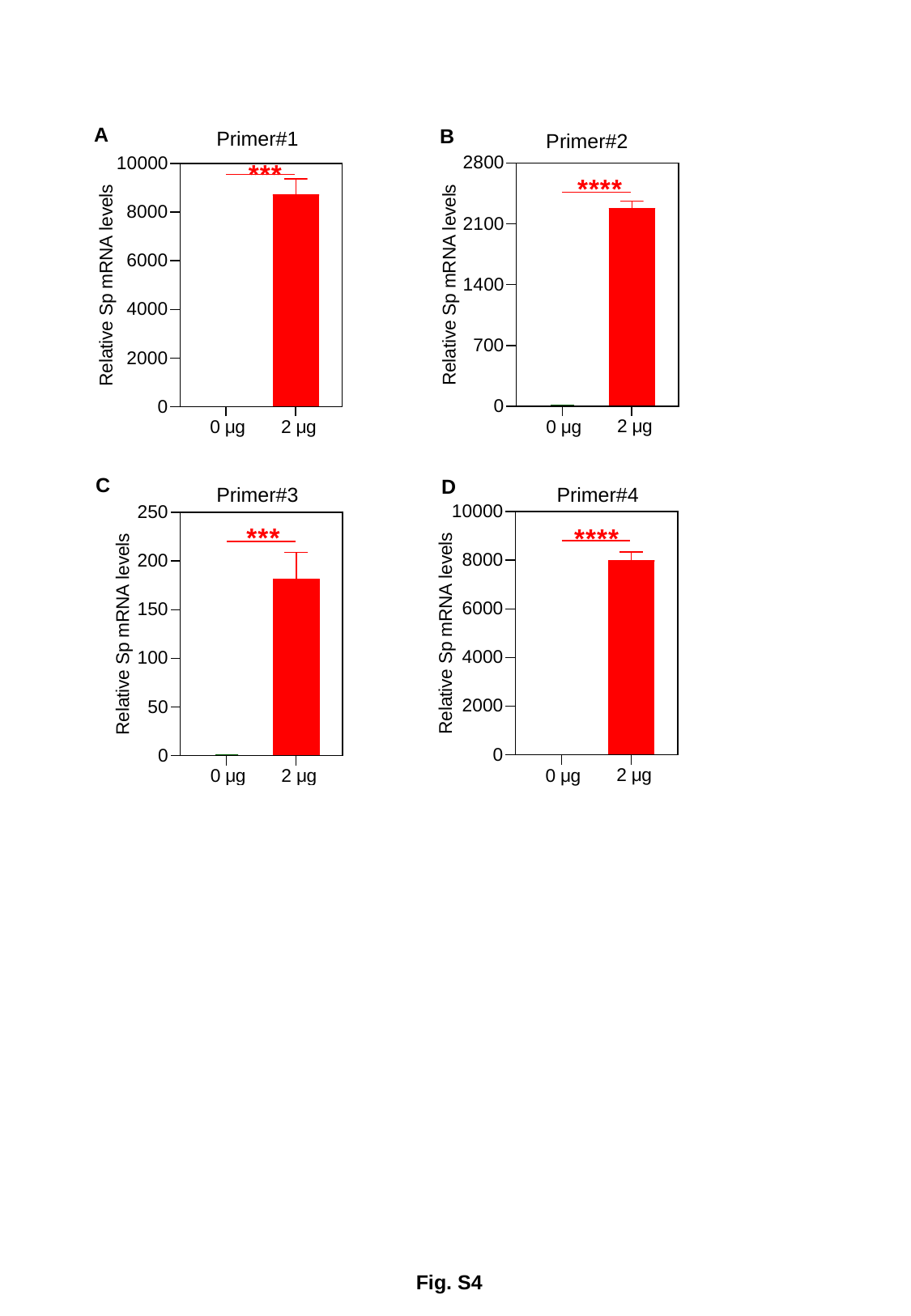

A
B
Primer#1
Primer#2
C
D
Primer#3
Primer#4
Fig. S4

## Slide 5
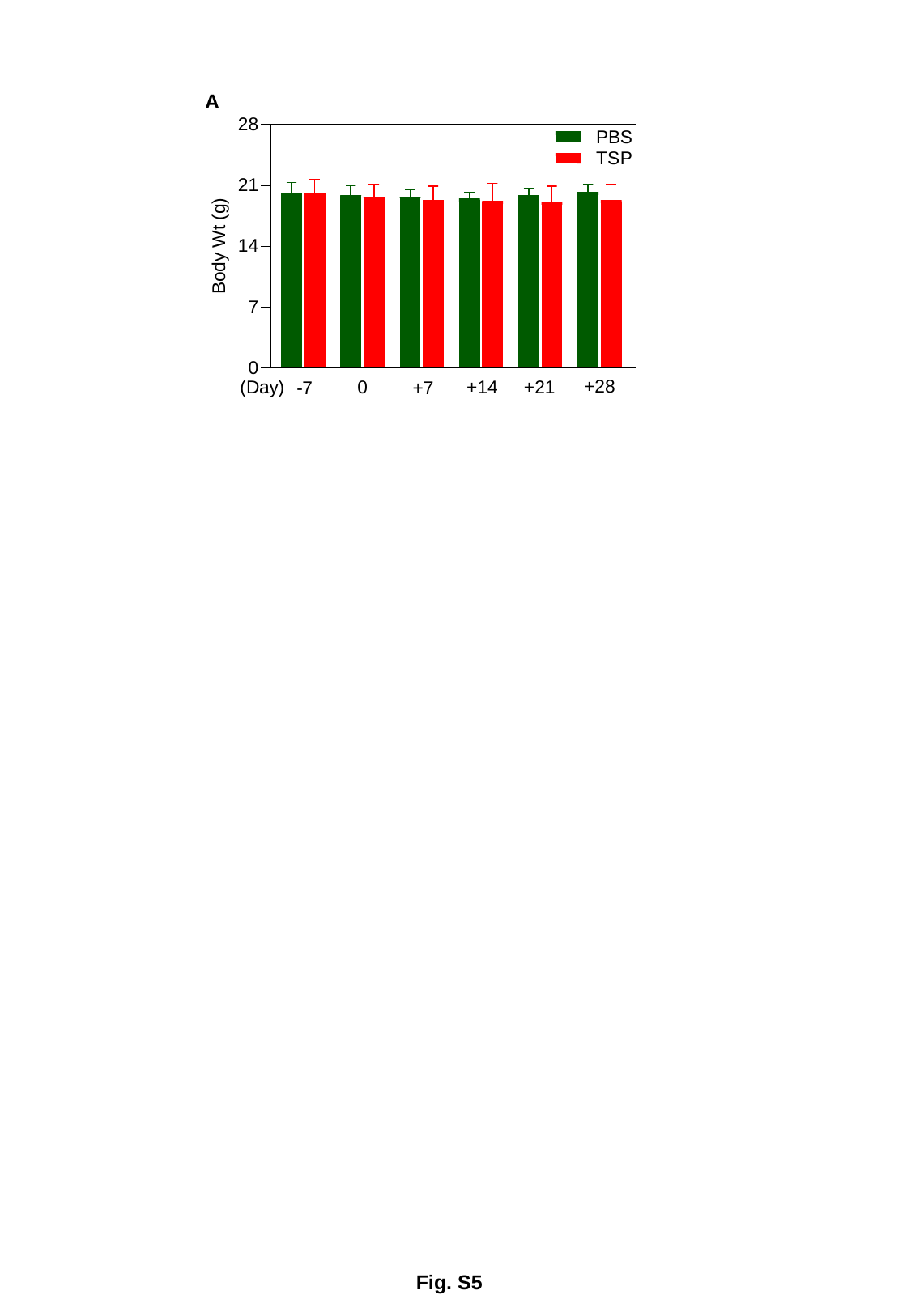

A
Fig. S5

## Slide 6
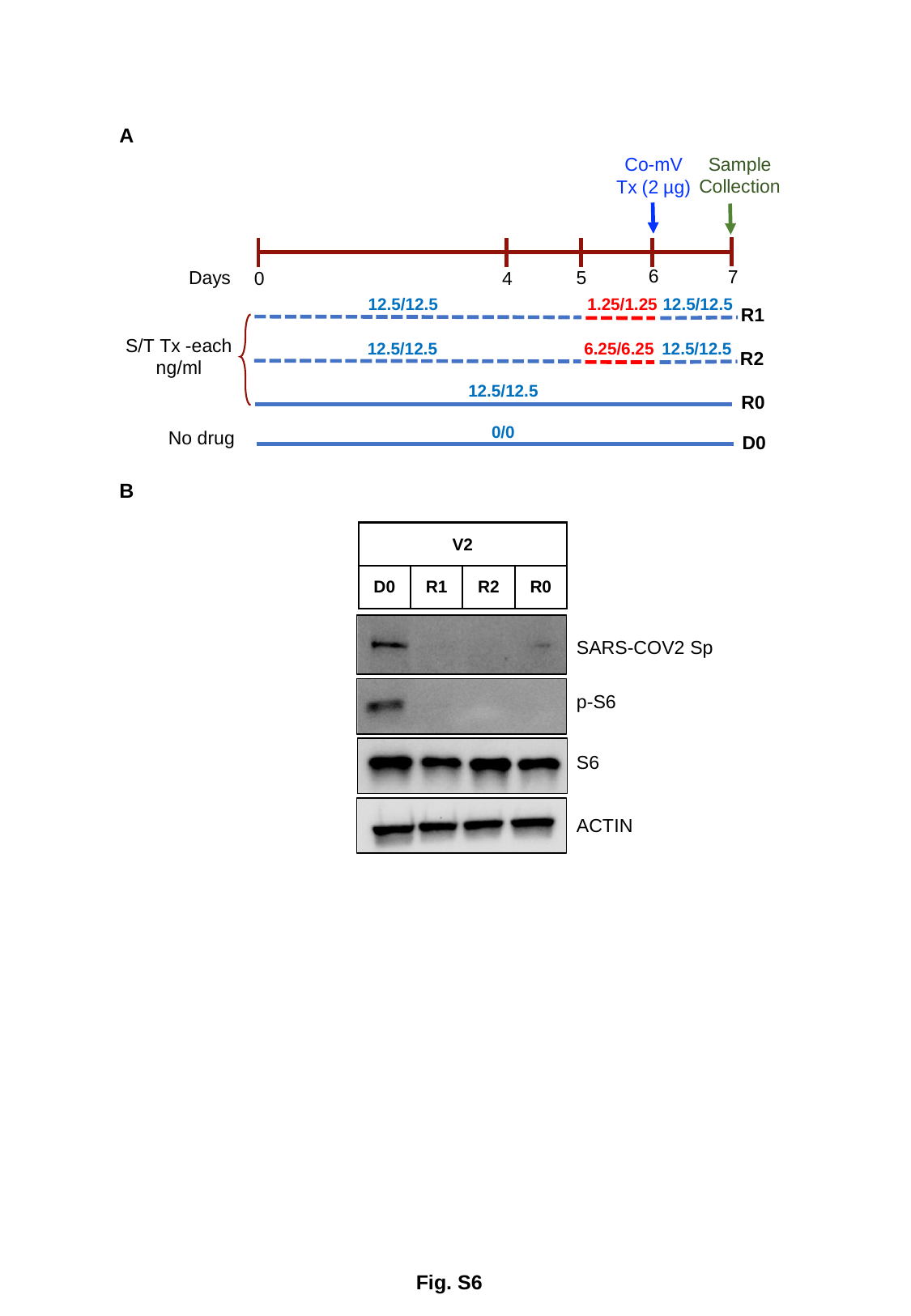

A
B
Fig. S6

## Slide 7
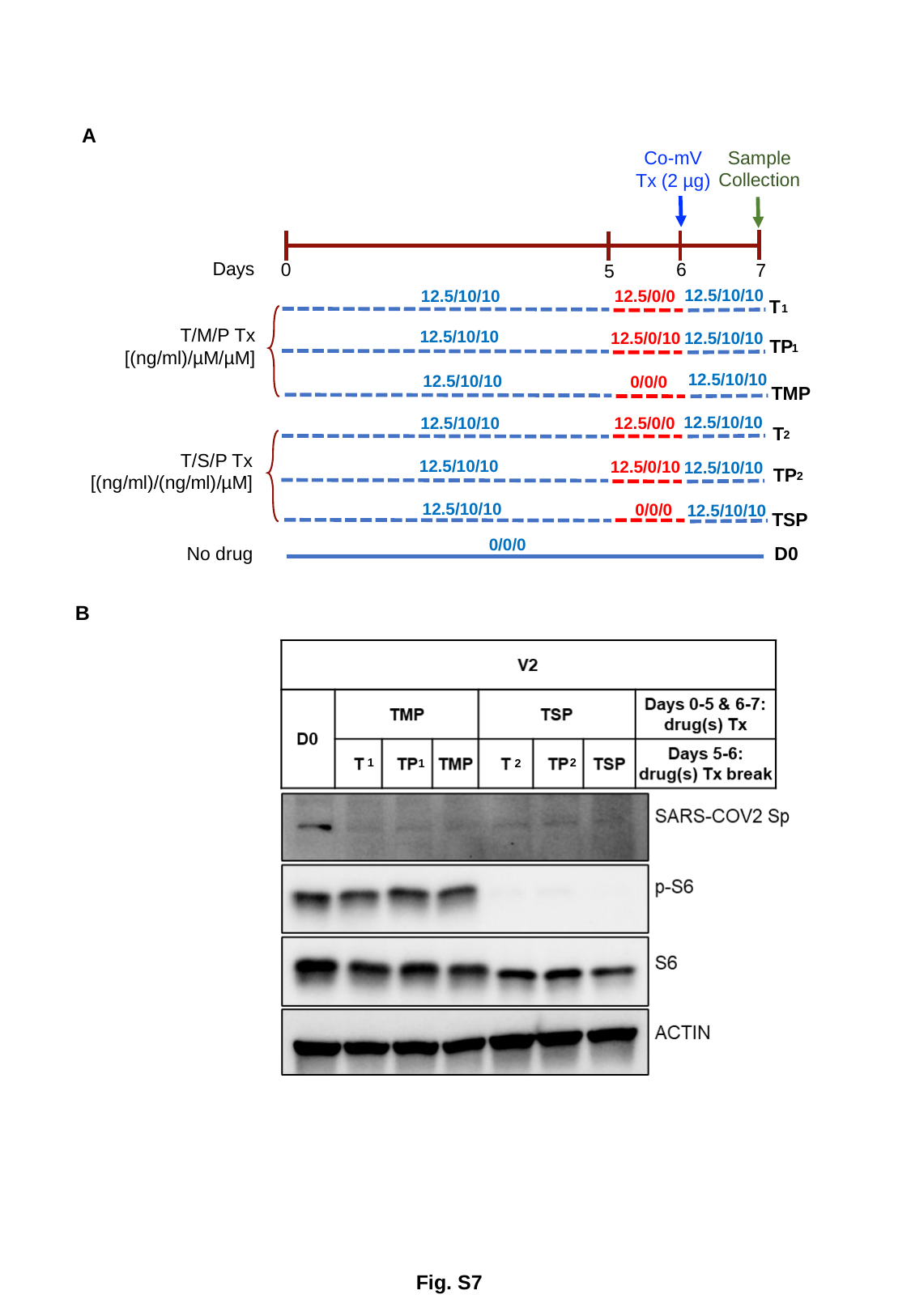

A
1
1
2
2
B
2
1
2
1
Fig. S7
